# Bioenergetic and metabolic aberrations in induced pluripotent stem cell-derived cardiomyocytes generated from a patient with Wolff-Parkinson-White syndrome caused a *PRKAG2* mutation

**DOI:** 10.3389/fcvm.2026.1719965

**Published:** 2026-04-10

**Authors:** Polina Baskin, Ifat Abramovich, Helena Milman, Ronen Ben Jehuda, Bella Agranovich, Mor Davidor, Valerie Buffard, Todd Herron, Ferhaan Ahmad, Michael Arad, Melanie Ricke-Hoch, Ofer Binah

**Affiliations:** 1 Department of Physiology, Biophysics and Systems Biology, Rappaport Faculty of Medicine and Research Institute, Technion, Haifa, Israel; 2 Perlmutter Metabolomics Center, Rappaport Faculty of Medicine and Research Institute, Technion, Haifa, Israel; 3 Division of Cardiovascular Medicine, Department of Internal Medicine, University of Iowa, Iowa City, IA, United States; 4 Department of Internal Medicine-Cardiology, University of Michigan, Ann Arbor, MI, United States; 5 Heart Institute, Sheba Medical Center, Faculty of Medicine, Tel-Aviv University, Tel Aviv, Israel; 6 Department of Cardiology and Angiology, Hannover Medical School, Hannover, Germany

**Keywords:** bioenergetic and metabolic profiling, calcium transients, iPSC-derived cardiomyocytes(iPSC-CMs) from a Wolf-Parkinson-White (WPW) patient, the *PRKAG2* gene, transgenic mice carrying the *PRKAG2* thr400Asn (T400N) mutation (TGT400N), Wolf-Parkinson-White syndrome (WPW)

## Abstract

**Introduction:**

The *PRKAG2* gene encodes the AMPK (AMP-activated protein kinase) γ2 subunit, regulating cellular energy homeostasis. *PRKAG2* mutations such as R302Q are associated with familial Wolff-Parkinson-White syndrome and hypertrophic cardiomyopathy, leading to metabolic dysregulation and cardiac dysfunction. Accordingly, we hypothesized that *PRKAG2*^*R302Q*^ mutation is associated with cardiac bioenergetic/metabolic deficits, causing cardiac dysfunction.

**Methods:**

Using WPW patient' iPSC-derived cardiomyocytes (iPSC-CMs) and a murine model carrying a *PRKAG2* mutation, we investigated the mutations-associated functional abnormalities.

**Results:**

We found in mutant iPSC-CMs compared to health iPSC-CMs, reduced glycolytic function and increased maximal mitochondrial respiration associated with elevated mitochondrial content, alongside increased glycogen accumulation, lipid storage and alterations in pathways related to redox regulation. Mutated murine hearts exhibited glycogen accumulation, altered glucose and lipid metabolism, elevated triacylglycerol levels and enhanced fatty acid oxidation pathways. Lipidomic and metabolomic analyses in both models revealed disrupted pathways linked to glucose and lipid metabolism. RNA-seq identified gene expression changes associated with redox regulation, mitochondrial function and hypertrophic signaling, aligned with the observed cellular and tissue-level dysfunction. Metformin treatment reduced mitochondrial content and respiration in mutant iPSC-CMs and significantly attenuated the arrhythmias.

**Discussion:**

These findings increase our understanding of *PRKAG2*-associated cardiomyopathy, and propose metformin as a novel modality for managing the metabolic and electrophysiological aberrations of this genetic disorder.

## Introduction

The *PRKAG2* gene encodes the 5′-adenosine monophosphate (AMP)–activated protein kinase (AMPK) subunit *γ*-2, which is involved in a variety of metabolic processes, such as regulating fatty acid and cholesterol biosynthesis, autophagy and glycogen degradation (1–4). The regulatory *γ* subunit of AMPK includes four cystathionine beta-synthase (CBS) domains, while each of the two CBS domains creates a binding site for AXP—Bateman domains (5). These domains enable the enzyme to detect the shifts in cell energy levels via AMP/ATP ratio, thus regulating catabolic and anabolic processes depending on the cell's current energetic status. The most common mutations in the *PRKAG2* gene are located within the CBS domains (e.g., *R302Q* in CBS1, H383R, *T400N* in CBS2 and *R531G* in CBS3), or in the linker sections (e.g., *N488I*) (6–9) thus interrupting the ability of AXP to bind the Bateman domains, leading to impaired regulation of the critical metabolic enzyme—AMPK.

Normal myocardial function requires proper energy generation and utilization, and relies on a series of complex metabolic processes. Structural and functional deficiencies can result in heart muscle dysfunction when the metabolic processes fail to work correctly or effectively. Thus, autosomal dominant mutations in the *PRKAG2* gene in humans lead to cardiac hypertrophy, ventricular pre-excitation—Wolff-Parkinson-White syndrome (WPW), a progressive conduction system disease, and vacuolar glycogen accumulation in cardiomyocytes (1, 8, 10–12). Despite extensive research on cardiomyopathies in recent years, the contribution of metabolic defects to the disease pathology remains unclear. Metabolic abnormalities, such as energetic deficiency, may impair myocardial force generation and relaxation, force transmission, electrophysiological function and overall cell survival. Based on the critical role of AMPK in major metabolic pathways, we tested the hypothesis that the *PRKAG2^R302Q^* mutation is associated with cardiac bioenergetic and metabolic deficits, thus causing cardiac dysfunction. The hypothesis was tested in induced pluripotent stem cell (iPSC)-derived cardiomyocytes (iPSC-CMs) from a patient with WPW (compared to isogenic control and healthy volunteers), as well as in a WPW mouse model. In support of the hypothesis, we found that the mutant iPSC-CMs (from now onwards, **CMs**) exhibit significantly lower glycolysis rates compared to isogenic control and healthy CMs. The abnormal glycolysis is likely compensated by increased mitochondrial activity. These metabolic alterations were mirrored in transgenic *PRKAG2*-mutated mice hearts, which exhibited glycogen accumulation and disrupted lipid metabolism, consistent with findings in *PRKAG2*-mutant CMs. Metabolomic analyses of transgenic mouse hearts showed altered glucose and lipid pathways, including increased triacylglycerol (TAG) levels and shifts toward fatty acid oxidation. Since the AMPK activator metformin serves as a mitochondrial respiratory chain complex I inhibitor in supra-pharmacological concentrations (13–16), we demonstrated that 24-hour metformin treatment attenuated the arrhythmias in *PRKAG2*-mutant CMs, suggesting abnormal mitochondrial function contributes to the impaired electrophysiological properties associated with the *PRKAG2^R302Q^* mutation.

## Methods

### The WPW patient and iPSCs generation

A dermal biopsy was obtained during pacemaker battery replacement in a 60-year-old male carrying the *PRKAG2*^R302Q^ mutation. The patient originally had cardiac hypertrophy, but following atrioventricular (AV) block and years of ventricular pacing, developed systolic dysfunction, in the form of hypokinetic hypertrophic cardiomyopathy (HCM), as previously described (7). The donor signed a consent form according to approval #7603-09-SMC of the Helsinki Committee for Experiments on Human Subjects at Sheba Medical Center, Ramat Gan, Israel. Several clones of mutant iPSCs were generated from the obtained biopsy and used to generate the isogenic control, as previously described (7). In addition, this study included a healthy clone previously characterized and described (7, 17, 18).

#### iPSCs culture and differentiation

iPSC differentiation into cardiomyocytes was performed according to the previously described directed differentiation by modulating Wnt/*β*-catenin signaling protocol (7). iPSCs were cultured on Matrigel (GFR, BD Biosciences, Franklin Lakes, NJ, USA)-coated 6-well plates in mTeSR1 medium (Stemcell Technologies, Vancouver, Canada) for 5–6 days. To initiate differentiation, cells were incubated with 1 mL/well Versene solution (Invitrogen, Life Technologies, Woburn, MA, USA) at 37 °C for 5 min and seeded on Matrigel-coated 12-well plates, in mTeSR1 medium. The medium was replaced daily, and, after 2 days when the monolayer of cells reached 100% confluence, the medium was changed to RPMI supplemented with B27 minus insulin (Invitrogen, Life Technologies, Woburn, MA, USA) containing 10 μM CHIR99021; this day was counted as day 1 of differentiation. On the next day (day 2 of differentiation), the medium was changed to RPMI supplemented with B27 minus insulin. On day 4, the medium was changed to RPMI supplemented with B27 minus insulin, containing 10 μM of IWP-4. On day 6, the medium was changed to RPMI supplemented with B27 minus insulin. Finally, from day 8 onwards, the medium was supplemented with RPMI with B27 complete supplement (Invitrogen, Life Technologies, Woburn, MA, USA). A total of no less than ten different differentiation protocols were utilized throughout this study.

### Mice experiments

All studies involving mice conformed to the *Guide for the Care and Use of Laboratory Animals* (NIH Publication No. 85-23, revised 1996) and were approved by the University of Iowa Institutional Animal Care and Use Committee (IACUC) under protocol #9081881. The volume of our standard mouse cages is 9 L, so we were using a 33% chamber volume per minute flow rate for CO_2_ administered, until 1 min after breathing stops. Euthanasia was confirmed by cervical dislocation prior to any tissue harvest. In the present study we used two mouse lines: (1) Sixteen-week-old male transgenic FVB strain mice carrying the *PRKAG2* Thr400Asn (T400N) mutation (TG^T400N^), previously constructed and described by Banerjee et al; (2) wild-type (WT) mouse line (19). Hearts were stored in liquid N_2_ immediately after sacrifice, and sent to the Technion on dry ice.

#### Generation of transgenic mice (TG^T400N^)

A T400N cDNA was generated from human cardiac RNA by PCR mutagenesis, and inserted into a pBluescript based vector with the mouse *α*-myosin heavy chain (*α*MHC) promoter, a highly active cardiac myocyte specific promote. The transgenic vector was linearized with Bam HI, size-fractionated, purified, and microinjected into fertilized FVB mouse oocytes at the University of Pittsburgh Transgenic and Chimeric Mouse Facility. Transgenic founders were identified by Southern blot analyses. Offspring of founders were genotyped by PCR amplification of the transgene using two sets of primer pairs—SPKG2 (5′-CCGCTCCTCCTCCAAAGAGT-3′) and ASPKG3 (5′-GCAATGTTGTGGTACGTTCC-3′) both within the *PRKAG2* cDNA; and MHC F1 (5′-CGGCACTCTTAGCAAACCTC-3′) within the vector backbone 5′ of the cDNA, and MHC R1 (5′-TTCTGGCTGGCATTTTTCTT-3′) within the cDNA.

#### The hemodynamic features of the transgenic mice

As reported by Banerjee et al. (19), at 16-week age (age of sacrifice) the functional parameters in WT and transgenic mice (TG^T400N^) were as follows (*n* = 4–7/group): Left ventricular anterior wall thickness (mm; LVAWS): 0.89 ± 0.03 and 1.64 ± 0.20*; Left ventricular End Diastolic diameter (mm, LVEDD): 3.17 ± 0.11 and 4.66 ± 0.32*; Fractional Shortening (FS%): 48 ± 3 and 16 ± 2*; Heart rate (bpm, HR): 506 ± 28 and 416 + 38. *p < 0.01 vs. WT.

### Measurements of extracellular acidification rate (ECAR) and oxygen consumption rate (OCR)

ECAR and OCR were measured using the Seahorse XFe96 Analyzer equipped with an optical fluorescent oxygen/hydrogen sensor (Agilent, Santa Clara, CA, USA). CMs were first dissociated using trypsin-EDTA solution (0.25% trypsin, 0.02% EDTA; Biological Industries, Kibbutz Beit-Haemek, Israel) and seeded on a Matrigel-coated (GFR, BD Biosciences, Franklin Lakes, NJ, USA) Seahorse 96-well plate at a density of 60,000CMs per well. Prior to measurements, the culture medium was changed to a glucose-free, L-glutamine-free, phenol red-free, sodium pyruvate-free, and sodium bicarbonate-free DMEM medium (D5030, Sigma Aldrich, St. Louis, MO, USA) supplemented with 2 mM glutamine for the Glycolysis Stress Test, or 1 mM pyruvate, 10 mM glucose, and 2 mM glutamine for the Mito Stress Test. The medium was titrated to pH 7.4 at 37 °C. The cells were incubated with 180 μL of the appropriate medium per well for at least 60 min in a CO_2_-free incubator at 37 °C for equilibration.

#### Glycolysis stress test (ECAR)

ECAR measurements were performed at baseline and following sequential injections of 10 mM glucose, 4 μM oligomycin (ATP synthase inhibitor), 50 mM 2-deoxy-glucose (2DG, a glucose analog). These measurements allowed us to determine: (1) *Glycolysis rate*: calculated by subtracting the baseline ECAR from the ECAR after glucose injection; (2) *Glycolytic capacity*: determined by subtracting the baseline ECAR from the ECAR after oligomycin injection; (3) *Glycolytic reserve*: Calculated by subtracting the ECAR after glucose injection from the ECAR after oligomycin injection.

#### Mito stress test (OCR)

OCR measurements were performed at baseline and following sequential injections of 4 μM oligomycin, 5 μM carbonyl cyanide-4-(trifluoromethoxy) phenylhydrazone (FCCP, a mitochondrial uncoupler), and 0.5 μM Antimycin A and 0.5 μM Rotenone (complex III and complex I inhibitors, respectively). These measurements allowed us to assess: (1) *ATP-linked respiration*: calculated by subtracting the OCR after oligomycin injection from the baseline OCR; (2) *Maximal respiration:* determined by subtracting non-mitochondrial respiration (Antimycin A and Rotenone OCR) from the FCCP-induced OCR; (3) *Mitochondrial OCR:* calculated by subtracting non-mitochondrial respiration from the basal OCR. All reagents were sourced from Sigma-Aldrich (St. Louis, MO, USA). The data was normalized to protein content measured using the modified Lowry method. This protocol enabled the comprehensive assessment of both glycolytic and mitochondrial function in CMs, providing information regarding their metabolic state under different experimental conditions.

### Measurement of mitochondrial membrane potential

CMs (0.5 × 10^5^) were obtained from a WPW patient and a healthy male donor. In addition, isogenic control CMs were generated from the patient cells (7). CMs from the three clones were seeded onto a 24-well plate (Nunclon Delta Surface, Thermo Scientific, Roskilde, Denmark) and grown for one week in RPMI culture medium (Invitrogen, Life Technologies, Carlsbad, CA, USA) with B27 supplement (Invitrogen, Life Technologies, Carlsbad, CA, USA). On the day of the experiment, the medium was replaced with serum-free EB medium supplemented with 200 nmol/L Mitotracker Green (MTG) (Invitrogen, Thermo Fisher Scientific) and 20 nmol/L Tetramethyl Rhodamine Ethyl Ester (TMRE) (Thermo Fisher Scientific) for 45 min at 37 °C. Beating CMs were imaged using the Zeiss Cell Discoverer 7 with a Plan-Apochromat 20×/0.7NA objective with a 1× tube lens and an Orca Flash 4.0 V3 CMOS mono-camera using the quadband filterset (emission filters 425/30 + 514/30 + 592/25 + 709/100), for MTG—LED excitation 493 nm, emission filter 525/50; for TMRE—LED excitation 575 nm, emission filter 605/70 as the quadband filterset resulted in crosstalk between the channels. The analysis was performed using the Zeiss Zen software (version 3.5, Carl Zeiss Microscopy GmbH, Jena, Germany). The ratio of TMRE intensity over MTG area/intensity was calculated to represent the mitochondrial activity rate.

### LC-MS-based metabolomics and lipidomics

#### Intracellular metabolite extraction

CMs were dissociated using trypsin 0.25% EDTA 0.02% solution (Biological Industries, Kibbutz Beit-Haemek, Israel) and washed twice with ice-cold PBS. For polar metabolomics profiling, Intracellular metabolites were extracted using cold (−20 °C) metabolite extraction solution consisting of methanol (Merck, 106035), acetonitrile (Merck, 100029) and water at a ratio of 5:3:2 respectively. For lipidomics profiling, Intracellular metabolites were extracted using Butanol (Merck, 1.01988) and Methanol (Merck, 106035) at a ratio of 1:1. For both analyses, cell extracts were centrifuged at 13,000 g for 10 min at 4 °C, and the supernatants were kept in −80 °C until Liquid chromatography–mass spectrometry (LC–MS) analysis.

#### Tissue metabolite extraction

TG^T400N^ and WT mice were sacrificed at the age of 16 weeks. The lower 2/3 of the frozen hearts tissues (apex) were weighing between 30 ± 2 mg were added to CK14 homogenizing tubes containing 1.4 mm ceramic beads (Bertin Corp, P000926-LYSK0-A) which were prefilled with 800 µL of cold (−20 °C) metabolite extraction solvent consisting of methanol (Merck, 106035), acetonitrile (Merck, 100029) and water at a ratio of 5:3:2. Samples were homogenized at 4 °C in a Precellys 24 tissue homogenizer (Bertin Corp, P002391-P24T0-A.0). Homogenization conditions were set to three cycles, 30 s each, at 6,500 rpm with a 30-second gap between each of the cycles to preserve low temperature. Homogenates were centrifuged at 18,000 g for 15 min at 4 °C. The supernatant was then collected in microcentrifuge tubes and centrifuged again under the same conditions. The cleared supernatants were transferred to glass HPLC vials (Agilent, 8010-0542) and kept at −80 °C until LC-MS analysis.

#### Liquid chromatography-mass spectrometry analysis

Polar metabolites analysis was performed as previously described (20). A Q-Exactive Orbitrap Mass Spectrometer (Thermo Scientific) was used together with a Thermo Scientific UltiMate 3000 HPLC system. HPLC setup consisted of a ZIC-pHILIC column (SeQuant, 150 × 2.1 mm, 5 μm, Merck KGaA) with a ZIC-pHILIC guard column (SeQuant, 20 × 2.1 mm). The aqueous mobile-phase solvent was 20 mM ammonium carbonate plus 0.1% ammonium hydroxide solution and the organic mobile phase was acetonitrile. The metabolites were separated over a linear gradient from 80% organic to 80% aqueous for 15 min. The column temperature was 45 °C, the flow rate 200 μL/min, and the running time 27 min. All metabolites were detected across a mass range of 75–1,000 m/z using the Q-Exactive mass spectrometer at a resolution of 35,000 (at 200 m/z) with electrospray ionization and polarity switching mode. Lock masses were used, and the mass accuracy obtained for all metabolites was below 5 p.p.m. Data were acquired with Thermo Xcalibur software. The peak area of the different metabolites was determined using Thermo TraceFinder 4.0 software. The metabolites were identified by the exact mass of the singly charged ion and known retention time on the HPLC column. Commercially available standard compounds were analyzed previously to determine ion masses and retention times on the ZIC-pHILIC column. Intracellular and extracellular metabolites were normalized by the modified Lowery method to quantify protein content.

Lipids analysis was performed using Vanquish Flex HPLC (Thermo Fisher Scientific) coupled to Orbitrap Exploris 240 mass spectrometer (Thermo Fisher Scientific). Lipids were separated on an Acclaim C30 column (100 × 2.1 mm; 3.0 µm; Thermo Fisher Scientific) maintained at 60 °C. The mobile phases consisted of 60:40 ACN: H_2_O with 10 mM ammonium formate and 0.1% formic acid (A) and 90:10 IPA:ACN with 10 mM ammonium formate with 0.1% formic acid (B). The gradient was as follows: 0–3 min 30% (B); 3–5 min 43% (B); 5–5.1 min 55% (B); 5.1–8 min 60% (B); 8–18 min 65% (B); 18–24 min 85% (B); 24–26 min 100% (B); and 26– 33 min 30% (B). Sample temperature was maintained at 6 °C in the autosampler and 5 µL of sample were injected into the LC-MS instrument. Thermo Orbitrap Exploris 240 MS instrument was operated in both positive and negative polarities, over the following mass range 120–1,700 m/z for both polarities (positive) at resolution 120,000. Data- dependent fragmentation (dd-MS/MS) was carried out for each polarity using dynamic exclusion, cycle time of 1.5 s using normalized collision energy and RES of 30,000. Scan range mode was defined to “Define First Mass”. Lipid identification was performed with LipidSearch 5.0 (Thermo Fisher). Lipids were annotated at the species level. The main parameters were precursor tolerance: 5 ppm, product tolerance: 5 ppm, and product ion threshold: 5%. All lipids identified were peak-aligned. The peak alignment method was set as the mean. The retention time deviation was set as 0.1 min. The peak filtering was set as New Filter, top rank, all isomer peak, and the identification level was selected as “A”, “B” or “C”. The resulting data were extracted and lipid molecules with relative standard deviation (RSD) > 30% in the pool samples were removed. Lipid molecules were deleted in cases of >30% missing data based on that extracted by LipidSearch™.

### RNA-seq

#### RNA extraction and quality control

RNA extraction from 10^6^ CMs was carried out using the “ReliaPrep™ RNA Cell Miniprep System Kit” (Promega, Fitchburg, WI, USA) according to the manufacturer's instructions. Quality control for total RNA was performed using TapeStation (Agilent). The RINe value of all samples was in the range of 8.5–9.6, indicating that all samples were of good quality.

#### Library prep and data generation

Twelve RNA-seq libraries (NEBNext UltraII Directional RNA Library Prep Kit for Illumina, cat. no. E7760) were produced according to the manufacturer's protocol using 800 ng total RNA. mRNAs pull-up was performed using Magnetic Isolation Module (NEB, cat. no. E7490). All 12 libraries were mixed into a single tube with equal molarity. The RNA-seq data was generated on Illumina NextSeq500, 75 cycles, high-output mode (Illumina, cat. no. FC-404-2005).

#### NGS QC, alignment and counting

Quality control was assessed using Fastqc (v0.11.5), reads were trimmed for adapters, low quality 3′ and minimum length of 20 using CUTADAPT (v1.12); 80 bp single-end reads were aligned to human reference genome (Homo_sapiens.GRCh38.dna.primary_assembly.fa from ENSEMBL) and annotation file (Homo_sapiens.GRCh38.92.gtf downloaded from ENSEMBL) using STAR aligner (v2.6.0a). The number of reads per gene was counted using Htseq (v0.9.1).

#### Descriptive analysis

Statistical analysis was performed using DESeq2 R package (version 1.20.0) (Genome Biology 2014 15:550). The similarity between samples was evaluated within DESeq2 package using correlation matrix, shown as heatmap and principal component analysis (PCA). The latter was drawn from the 500 most variable genes.

#### Interactions, pathways and networks analysis

The DEGs lists were analyzed using the Ingenuity Pathway Analysis (IPA, version 9.0, QIAGEN, Redwood City, CA, USA).

### Action potentials recording and analysis

For action potential recordings, spontaneously contracting areas of the CM monolayer culture were mechanically dissociated to achieve the dispersion of cells into small clusters. These dispersed CMs were then plated onto Matrigel-coated glass coverslips (13 mm diameter) placed in 24-well plates. The coverslips were incubated at 37 °C, allowing a recovery period of 2 days before electrophysiological experiments were performed (7, 21–23). During experiments, the coverslips were continuously perfused at 37 °C with an external solution composed of the following (in mmol/L): 140 NaCl, 5.4 KCl, 1.8 CaCl_2_, 1 MgCl_2_, 10 glucose, and 10 HEPES, titrated to pH 7.4 with NaOH (310 mOsm). The patch pipette solution contained (mmol/L): 120 KCl, 1 MgCl_2_, 3 Mg-ATP, 10 HEPES, and 10 EGTA, titrated to pH 7.2 with KOH and adjusted to 290 mOsm with saccharose (all reagents were obtained from Sigma-Aldrich, St. Louis, MO, USA). An Axopatch 200B amplifier, Digidata 1322 or 1440 digitizer, and pClamp10 software (Molecular Devices, Sunnyvale, CA, USA) were used for data amplification, acquisition, and analysis. Signals were digitized at sampling rates of 4–10 kHz. Patch electrodes with resistances of 4–7 MΩ were pulled from borosilicate glass capillaries (Harvard Apparatus, Holliston, MA, USA). The electrophysiological recordings were analyzed to detect all peaks in the recorded signals, from which action potential parameters were calculated using dedicated MATLAB software (21). In these experiments, CMs were pre-treated for 24 h with 1 mM or 2.5 mM of the AMPK activator Metformin (1115-70-4, Cayman Chemical, Ann Arbor, MI, USA).

### Statistical analysis

Results are presented as mean ± SEM. Normality was assessed via Kolmogorov–Smirnov or Shapiro–Wilk tests. If passed, comparisons were performed using a One-Way or Two-way ANOVA test followed by the Holm–Sidak test, and t-test (for comparisons between two groups only). If normality was not assessed, Kruskal–Wallis nonparametric test was performed using GraphPad Prism 10 software (Boston, MA, USA). A value of *p* < 0.05 was considered statistically significant. For both studies, the results were tested after two iterations of outliers, excluding using mean ± 3SD.

## Results

### The glycolytic function in *PRKAG2*-mutant vs. healthy iPSC-CMs

**Figure 1:**
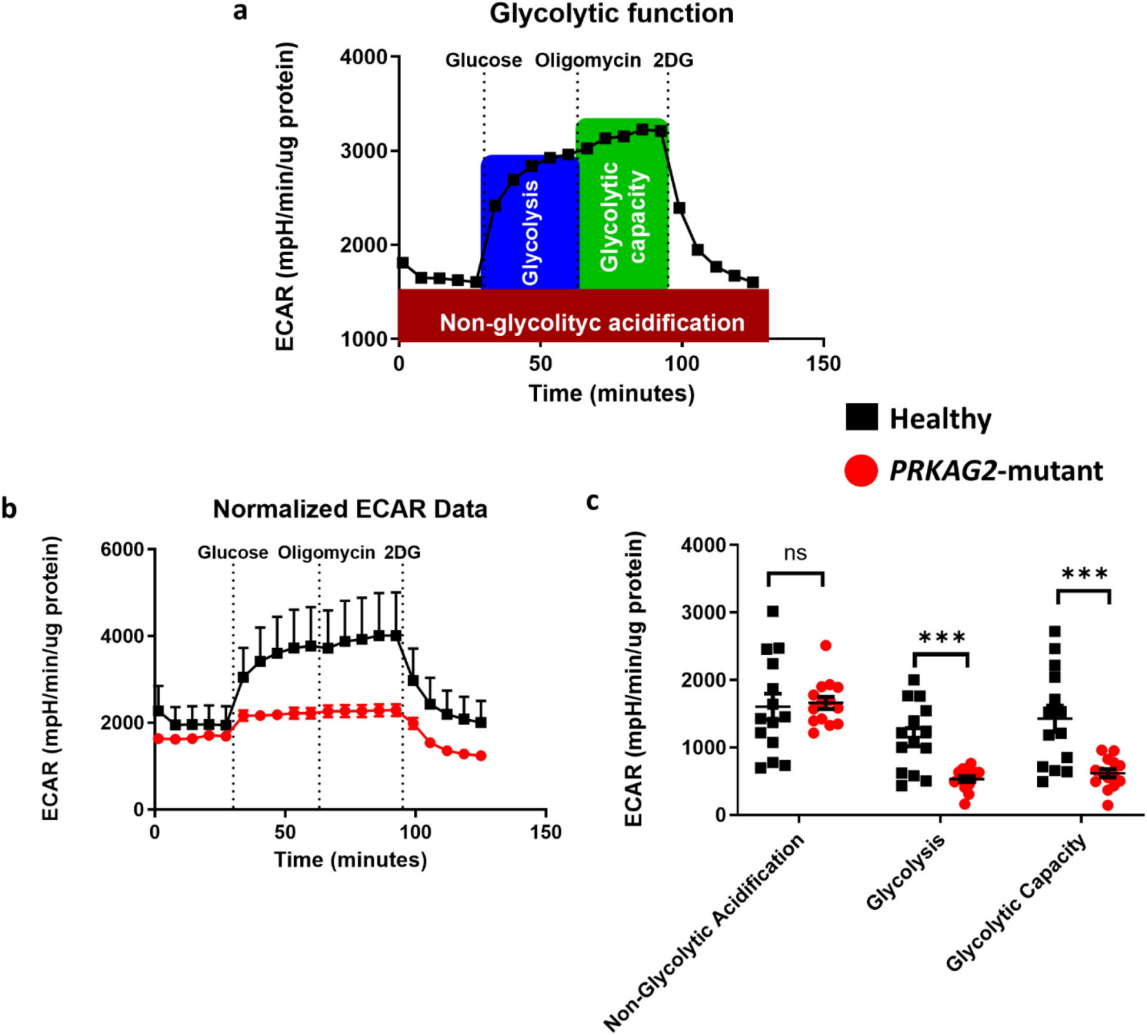
Glycolytic profile of *PRKAG2*-mutant and healthy iPSC-CMs (CMs) measured by XFe96 Seahorse metabolic flux analyzer. **(a)** Representative profile of glycolytic function in healthy CMs. Extracellular acidification rate (ECAR) is recorded during sequential, in-port additions of the glycolysis modulators: (1) Glucose, fuels glycolysis and thus leads to basal glycolysis and elevation of ECAR levels; (2) Oligomycin, an ATP Synthase inhibitor used to increase glycolysis dependency, leads to maximal ECAR values; (3) 2-DG, glucose-competitive inhibitor, reduces glycolysis-dependent ECAR levels to a minimum. **(b)** ECAR in *PRKAG2*-mutant (red) compared to healthy (black) CMs recorded during sequential additions of the glycolysis modulators: glucose, oligomycin and 2-DG. **(c)** Non-glycolytic acidification, glycolysis and glycolytic capacity rates calculated based on panel b. A significant decrease in glycolysis and glycolytic capacity rates was measured in *PRKAG2*-mutant compared to healthy CMs, while the non-glycolytic acidification rate was similar in these groups. ECAR values are normalized to μg protein measured using a modified Lowry protein assay. Sample sizes (*n*) refer to the number of wells analyzed in a 96-well Seahorse plate. Healthy, *n* = 14; *PRKAG2*-mutant, *n* = 14 CMs. Shapiro-Wilk test for normality was performed on data. An unpaired t-test was performed; NS = non-significant, ***P < 0.001.

Because AMPK is a key metabolic enzyme regulating cell energy and metabolism, we investigated whether the *PRKAG2* mutation affects glycolysis and oxidative phosphorylation in CMs. To measure the glycolytic function, we used the XFe96 Seahorse metabolic flux analyzer for extracellular acidification rate (ECAR) measurements during the glycolysis stress test (Glyco-stress test) (Figure 1). As shown in the representative Glyco-stress test results (Figure 1a), the first five measurements at the beginning of the experiment represent the non-glycolytic acidification rate, whereas glucose injection fuels glycolysis and leads to ECAR elevation representing the basal glycolysis. Injection of oligomycin, an ATP synthase inhibitor, increases glycolysis dependency as an energy source, therefore resulting in maximal ECAR values. Finally, 2-DG, a glucose-competitive inhibitor of glucose-6-phosphate, inhibits glycolysis completely, thereby reducing glycolysis-dependent ECAR levels to a minimum. Figure 1b shows representative results from a Glyco-stress test performed in *PRKAG2-*mutated (red) and healthy (black) CMs. In both groups, before the first glucose injection, ECAR values were stable and comparable. Glucose injection resulted in a significant ECAR increase in healthy CMs, while in the mutant CMs, only a slight increase was observed. Oligomycin caused an additional expected elevation, leading to maximal ECAR levels, in healthy CMs, but not in the *PRKAG2*-mutant CMs. Finally, 2-DG injection blocked glycolysis completely and reduced glycolysis-dependent ECAR levels to a minimum. In summary, these experiments demonstrate decreased glycolytic function in mutant compared to healthy CMs.

### The respiration profile of mutant, isogenic control and healthy iPSC-CMs

**Figure 2:**
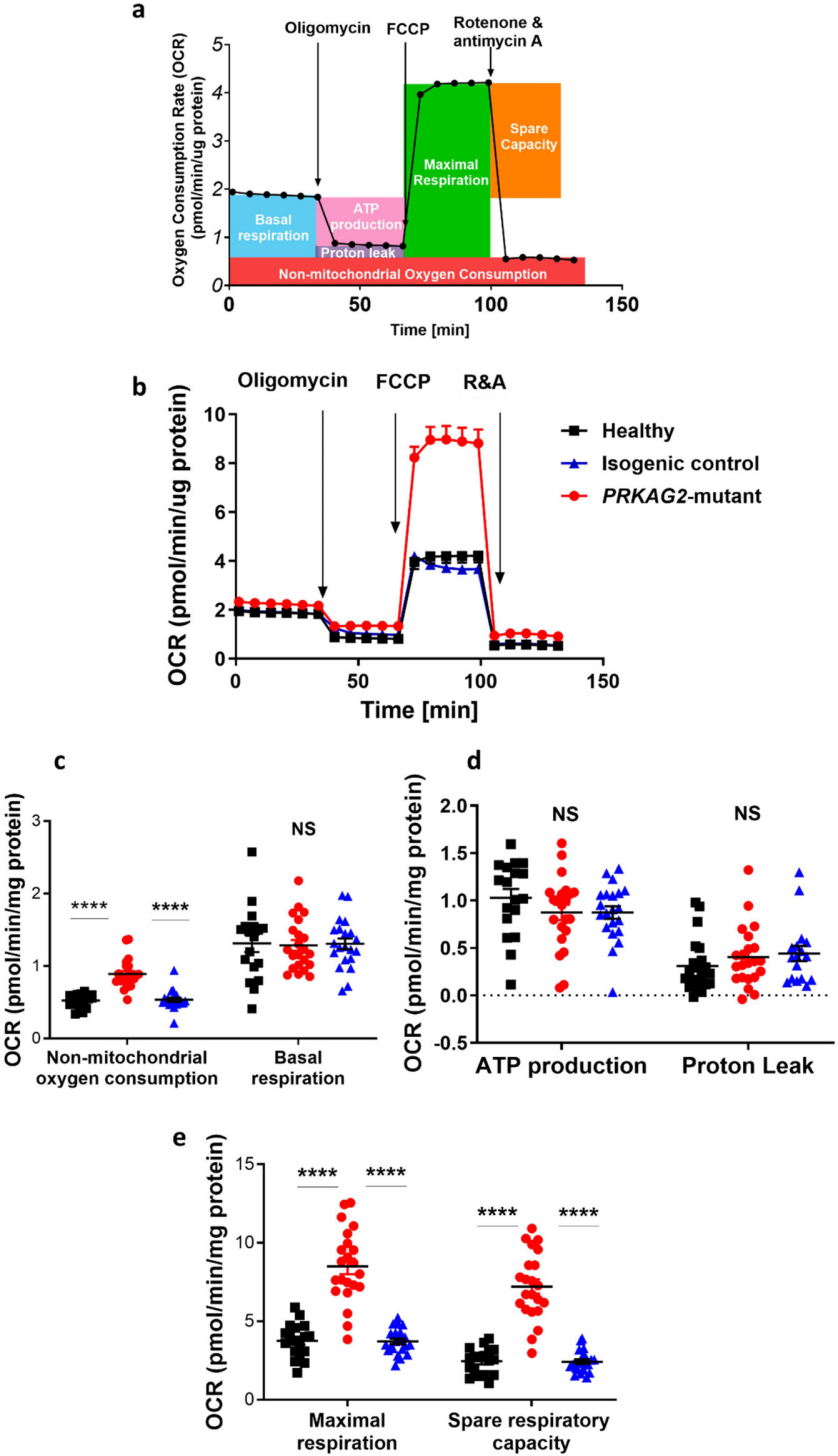
Respiration profile of healthy, *PRKAG2*-mutant, and isogenic control iPSC-CMs (CMs) by the XFe96 Seahorse metabolic flux analyzer. (**a**) Representative respirometer profile of healthy control iPSC-CMs. Oxygen consumption rate is recorded during sequential, in-port additions of mitochondrial modifiers: (1) Oligomycin, an ATP synthase inhibitor used to measure the oxygen consumption coupled to ATP synthesis; (2) FCCP, dissipates the mitochondrial proton gradient and stimulates maximal mitochondrial respiration capacity; (3) Rotenone and antimycin A, inhibitors of complex III and I, hence blockers of mitochondrial respiration. (**b**) Oxygen consumption rate (OCR) in mutant (red), isogenic control (blue) and healthy (black) CMs recorded during sequential additions of mitochondrial respiration modulators: Oligomycin, FCCP, and rotenone plus antimycin A. (**c–e**) Non-mitochondrial oxygen consumption, basal respiration, ATP production, proton leak, maximal respiration and spare respiratory capacity rates calculated based on plot B. A significant increase in non-mitochondrial oxygen consumption, maximal respiration and spare respiratory capacity measured in mutant, isogenic control and healthy CMs, while basal respiration, ATP production and proton leak rates are similar in all groups. OCR values are normalized to μg protein measured using a modified Lowry protein assay. Sample sizes (*n*) refer to the number of wells analyzed in a 96-well Seahorse plate. Healthy control, *n* = 18; mutant, *n* = 22; isogenic control, *n* = 21 CMs. Kruskal–Wallis test, followed by Dunn's multiple comparisons test.; NS = non-significant, ****p < 0.0001.

Following our findings that the *R302Q* mutation results in a decreased glycolytic rate (Figure 1), we investigated whether this mutation also affects the most prominent energy production process—oxidative phosphorylation. In this regard, we measured mitochondrial respiratory function, using the XFe96 Seahorse metabolic flux analyzer for oxygen consumption rate (OCR) measurements during the mitochondrial stress test (Mitostress test). The test was performed in mutant, isogenic control and healthy CMs (Figure 2). As shown by the representative Mitostress test results (Figure 2a), six consecutive OCR measurements were performed under basal conditions, followed by five OCR measurements performed after each sequential, in-port, addition of a specific mitochondrial modifier. The first modifier is oligomycin—an F_1_F_0_ ATP-synthase (Complex V in electron transport chain) inhibitor (24); since oligomycin blocks ATP production and interrupts the electron transport chain, its addition decreases the OCR. Oligomycin does not block mitochondrial respiration completely, as protons can still leak across the inner mitochondrial membrane independently of ATP-synthase. Thus, low mitochondrial oxygen consumption is still present after oligomycin injection. Hence, oligomycin enables the measurement of ATP synthesis-coupled OCR by subtracting the minimum OCR after oligomycin injection, from the last OCR measurement in basal conditions; and the proton leak-associated OCR by subtracting non-mitochondrial OCR from the minimum OCR measurement after oligomycin injection. The second injected modifier is FCCP, a mitochondrial uncoupler, which dissipates the mitochondrial proton gradient and prevents the electron transport chain from progressing (25). Therefore, the OCR increases to create a proton gradient, while FCCP prevents its production. Consequently, after FCCP injection, maximal mitochondrial OCR can be measured, and the spare respiratory capacity is calculated by subtracting the basal respiration OCR from the maximal OCR. Finally, the third injected modifier is a mixture of rotenone and antimycin A, electron transport chain complexes III and I inhibitors, respectively (26); together they completely block mitochondrial respiration, leading to decreased OCR. At this stage, there is no mitochondrial oxygen consumption, and the only measured OCR originates from a non-mitochondrial source. Thus, the injection of rotenone and antimycin A enables the measurement of non-mitochondrial OCR, which is crucial for calculating the previously described parameters.

Figure 2b shows representative results from a Mitostress test performed simultaneously in mutant (red), isogenic control (blue) and healthy (black) CMs. In all 3 groups, under basal conditions (6 measurements), OCR values were stable and comparable, and decreased similarly after oligomycin injection (5 measurements). The addition of FCCP resulted in similar OCR increases in isogenic control and healthy CMs (∼2-fold from basal OCR), whereas in the mutant CMs, FCCP had a dramatic effect; OCR increased ∼4.5 times from basal OCR and a∼2-fold increase compared to the response of isogenic control and healthy CMs. Finally, following the addition of rotenone and antimycin A, OCR drastically decreased in all 3 groups, attaining a similar lowest OCR level, which represents non-mitochondrial oxygen consumption only, since the process of electron transport chain is completely blocked. In summary, these experiments showed a significant increase in non-mitochondrial OCR in mutant, compared to CRISPR-corrected isogenic control and healthy CMs (Figure 2c). In addition, basal OCR was similar in all 3 groups, indicating a mutual starting respiratory status (Figure 2c). In agreement with the similarity in basal OCR among the groups, both components of ATP-linked, and proton leak-associated OCRs were also comparable (Figure 2d). Importantly, maximal mitochondrial respiration, calculated by subtracting non-mitochondrial respiration from the post-FCCP OCR, increased in the mutant, compared to both isogenic control and healthy groups. In agreement with maximal mitochondrial respiration results, spare respiratory capacity, calculated by subtracting basal respiration from the maximal OCR, was also increased in *PRKAG2*-mutant, compared to isogenic control and healthy CMs (Figure 2e). These findings indicate that maximal mitochondrial respiration and spare respiratory capacity were significantly increased in mutant CMs compared to isogenic control and healthy CMs, suggesting a difference in either mitochondrial content and/or activity among the different CM groups.

### The mitochondrial content and activity in mutant, isogenic control and healthy iPSC-CMs

**Figure 3:**
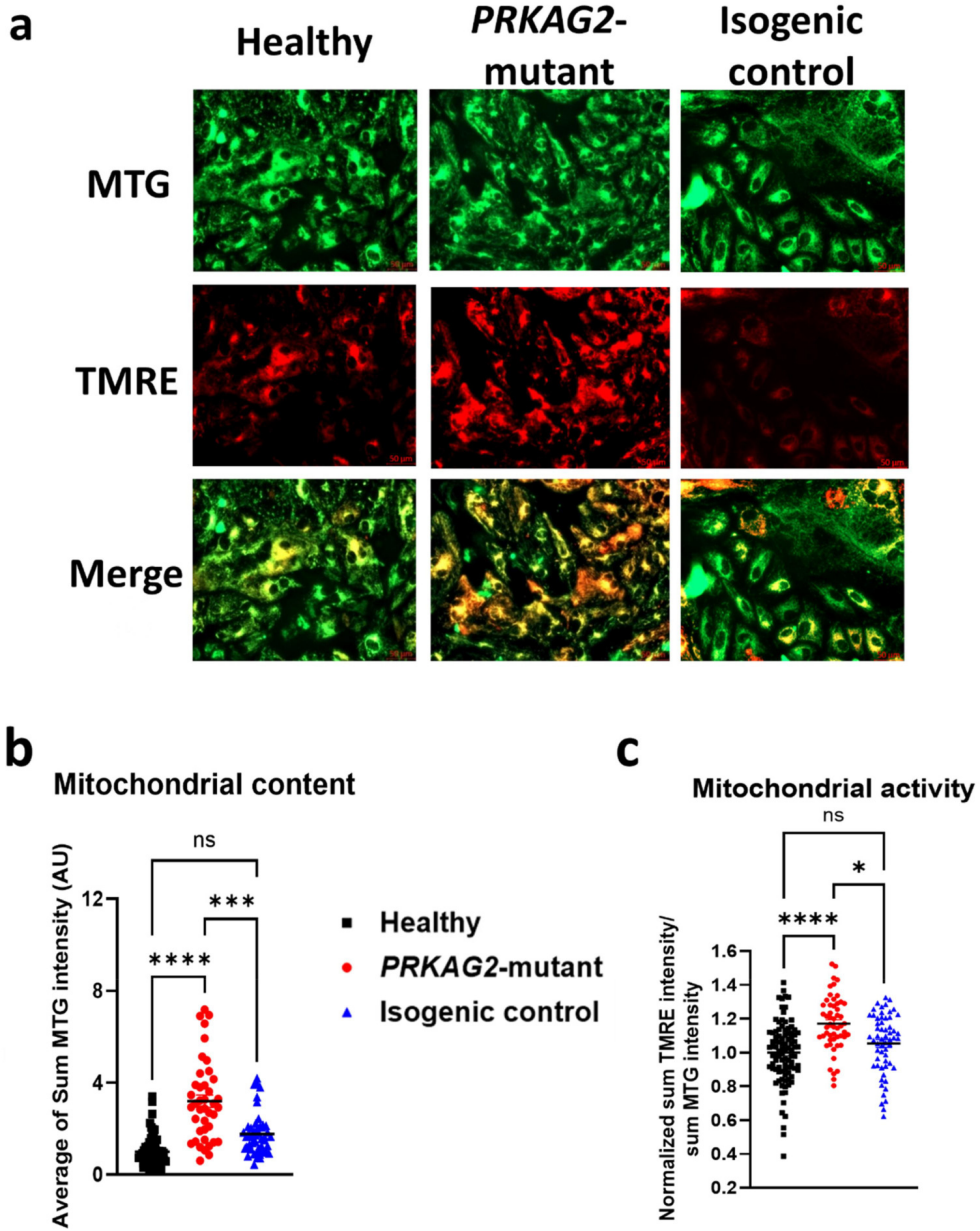
Mitochondrial content and activity in beating iPSC-CMs (CMs). (**a**) Representative confocal images of mitochondria in healthy (left), *PRKAG2-*mutant (middle), and isogenic control (right) CMs, simultaneously stained with 2 fluorescent dyes: MTG (green; *ΔΨ* independent), and TMRE (red; *ΔΨ* dependent). (**b**) Quantification of total mitochondrial content by MTG intensity. (**c**) Quantification of total mitochondrial activity calculated by the ratio of TMRE over MTG fluorescence (TMRE/MTG), of each image. The summary shows a significant increase in both mitochondrial activity and content in the mutant cells, compared to healthy and isogenic control. Cell Discoverer 7, Objective X20. Sample sizes (*n*) refer to image segments (defined areas within confocal images containing mitochondria). Multiple images were analyzed per well, and each image contained several segments. Healthy, *n* = 94; Mutant, *n* = 57; Isogenic control, *n* = 59. One-way ANOVA, ns = non-significant, *p < 0.05, **** *p* < 0.0001.

The mitochondrial respiration profile experiments demonstrated increased maximal respiration and spare respiratory capacity rates in mutant, compared to isogenic control and healthy CMs. To determine whether these changes are caused by an increased mitochondrial content and/or altered function in the mutant CMs, these parameters were measured in contracting CMs by acquiring live microscopy images using Cell Discoverer 7 (Figure 3a). Specifically, we simultaneously used MitoTracker Green (MTG) and tetramethylrhodamine ethyl ester (TMRE) fluorescent staining for labeling total and active mitochondria, respectively. Briefly, MTG accumulates in mitochondria independently of changes in mitochondrial membrane potential (*ΔΨ*_M_), while TMRE is sensitive to changes in *ΔΨ*_M_ (positively charged red-orange dye), and accumulates specifically in active mitochondria. Figure 3a shows representative mitochondria images of mutant, isogenic control and healthy CMs. As shown in Figure 3b, mitochondrial content was increased almost two-fold in the mutant compared to isogenic control and healthy CMs. Furthermore, mitochondrial activity (Figure 3c), calculated as a ratio of active mitochondrial intensity (TMRE intensity) over total mitochondrial content (MTG intensity), was increased in the mutant CMs by ∼20% compared to isogenic control and healthy CMs. In summary, mutant CMs demonstrate a significant increase in both mitochondrial content and activity, compared to isogenic control and healthy CMs, explaining the observed increased maximal respiration and spare respiratory capacity (Figure 2).

### Metabolic profiling of mutant and isogenic control iPSC-CMs, and mutated and WT mice

**Figure 4:**
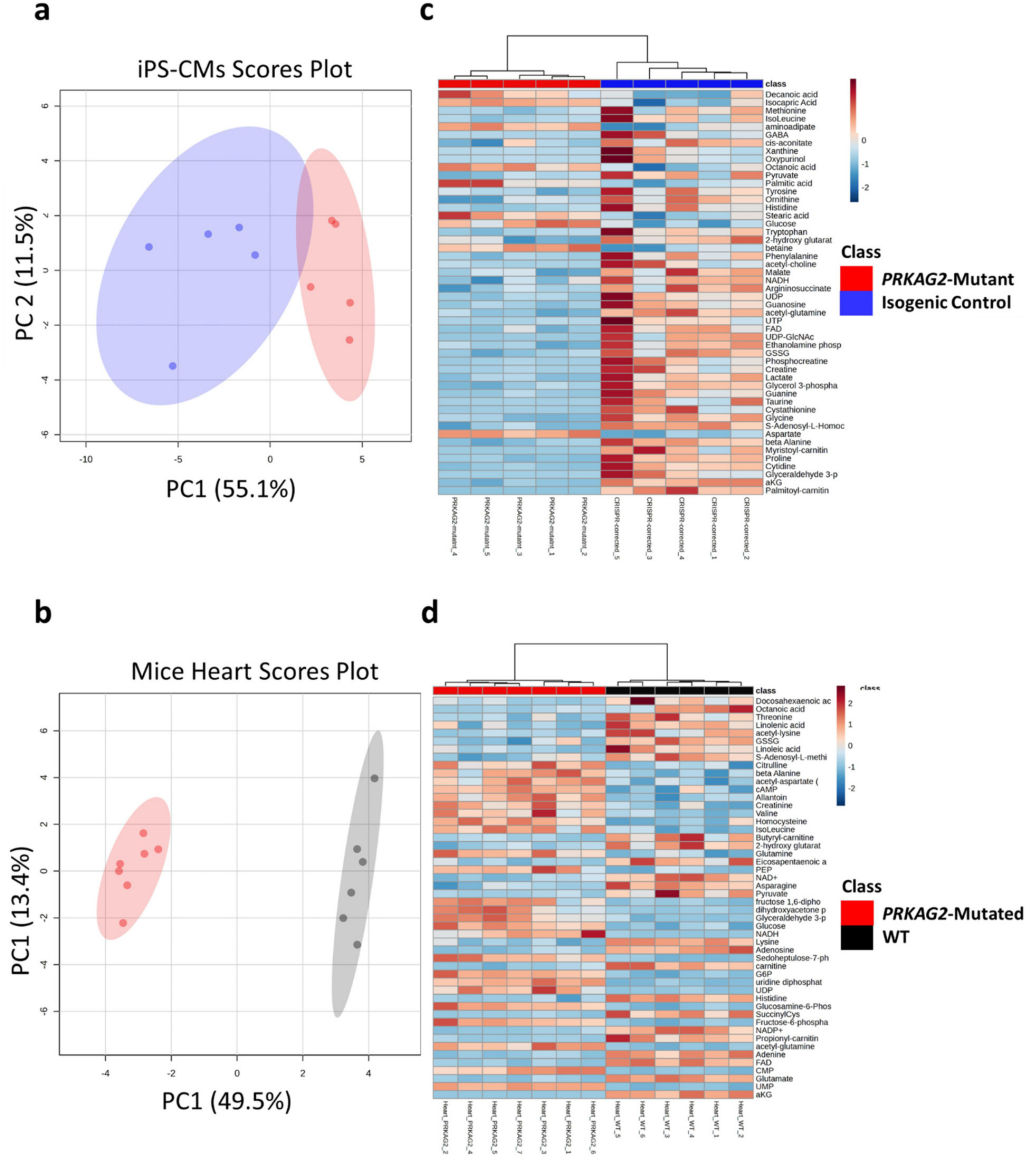
Principal component analysis and heatmap of metabolomic profiles in *PRKAG2* mutant and isogenic control iPSC-CMs (CMs), and in *PRKAG2* mutated and WT mice. (**a,b**) PCA of the complete metabolomic dataset comparing *PRKAG2* mutant and controls. (**a**) *PRKAG2*-mutant (red) vs. isogenic control (blue) CMs. (**b**) *PRKAG2*-mutated (red) vs. WT (black) mice hearts. (**c,d**) Heatmap of the top 50 differentially expressed metabolites in (**c**) *PRKAG2*-mutant vs. isogenic control CMs, and (**d**) *PRKAG2*-mutated vs. WT mice hearts. Metabolite abundance is represented by a color gradient (red = high, blue = low). The figure illustrates that the mutant CMs/mutated mice hearts exhibit distinct metabolic alterations compared to the control groups, especially in the top 50 metabolites visualized in the heatmaps. mutant CMs, *n* = 5; isogenic control CMs, *n* = 5; mutated mice hearts, *n* = 7; WT mice hearts, *n* = 6.

To investigate the metabolic consequences of the mutation, we performed a comprehensive metabolomic and lipidomic analysis in mutant and isogenic control CMs, as well as in mutant and WT mouse lower 2/3 heart tissue. First, Principal Component Analysis (PCA) was performed to assess the global metabolic differences. Whereas the PCA of the complete metabolomic dataset revealed a certain degree of separation between *PRKAG2^R302Q^*-mutant and isogenic control CMs (Figure 4a), there was also a partial overlap between the two groups. This overlap is likely caused since both CMs have identical genetic background and therefore have identical metabolic baseline despite the mutation and its correction. In contrast, Figure 4b shows a more distinct separation between *PRKAG2^T400N^*-mutated and WT mice hearts. The more prominent divergence may be related to chronic changes at the whole organ level (e.g., changes in a cell population; CMs vs. fibroblasts), that will not be evident in cultured iPSC-CMs. The heatmaps in Figures 4c,d highlight the top 50 differentially expressed metabolites in each model. Figure 4c shows the relative abundance of metabolites between *PRKAG2*-mutant and isogenic control CMs, revealing several metabolic changes despite the overlapping profiles in the PCA. Figure 4d presents the top 50 differentially expressed metabolites in *PRKAG2*-mutated vs. WT mice hearts, where more distinct alterations in metabolite levels are evident.

### Ingenuity pathway analysis (IPA) analyses

**Figure 5:**
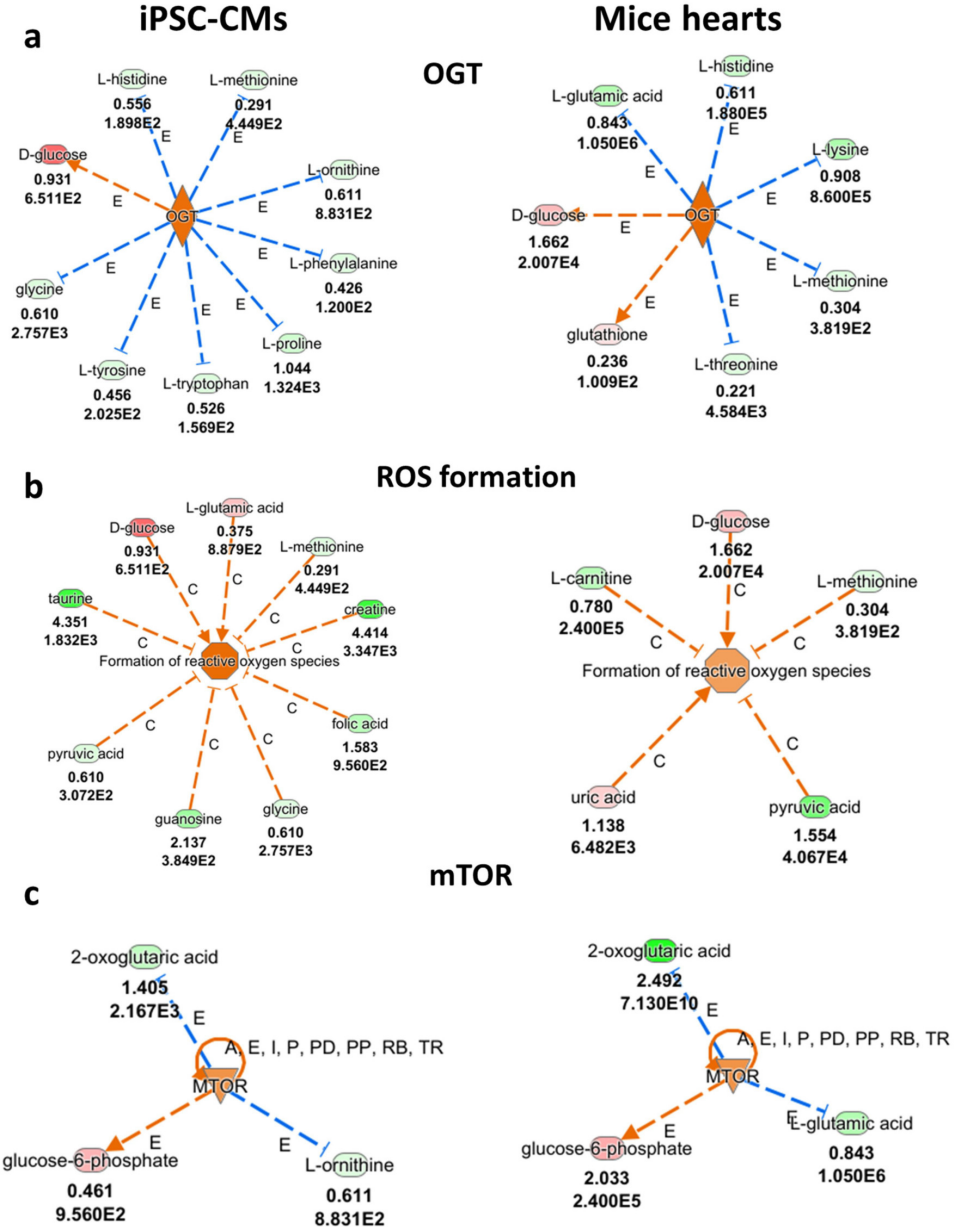
Metabolic pathways related to stress response and energy regulation in *PRKAG2*-mutant iPSC-CMs (CMs) and mice hearts, analyzed by ingenuity pathway analysis (IPA). (**a**) Increased O-GlcNAc Transferase (OGT) activity in mutant CMs and mice hearts is indicated by elevated levels of key metabolites, including D-glucose, L-glutamic acid and glutathione (red). These findings suggest a heightened cellular stress response. (**b**) Elevated reactive oxygen species (ROS) formation is observed in mutant CMs and mice hearts. Increased levels of L-carnitine, L-methionine, and glutathione (red), along with orange predictions, indicate increased oxidative stress. (**c**) Disruptions in the mTOR pathway are shown, with elevated levels of 2-oxoglutaric acid and glucose-6-phosphate (red) in both mutant CMs and mice hearts. Orange predictions indicate increased mTOR pathway activity, suggesting heightened energy regulation and biosynthetic processes. The upper numbers refer to log2(fold change), and the lower numbers represent the False Discovery Rate (FDR). In these visualizations, “E” represents experimentally observed changes in metabolite levels, while “C” denotes computational predictions, including inferred upstream regulators and pathway-level impacts. Central structures highlight key regulatory hubs or metabolic nodes significantly affected by the *PRKAG2* mutations R302Q and T400N. Blue and orange arrows indicate predicted activation (orange) or inhibition (blue) based on z-scores from IPA analysis. The curved arrows represent feedback loops or alternative pathway fluxes.

**Figure 6:**
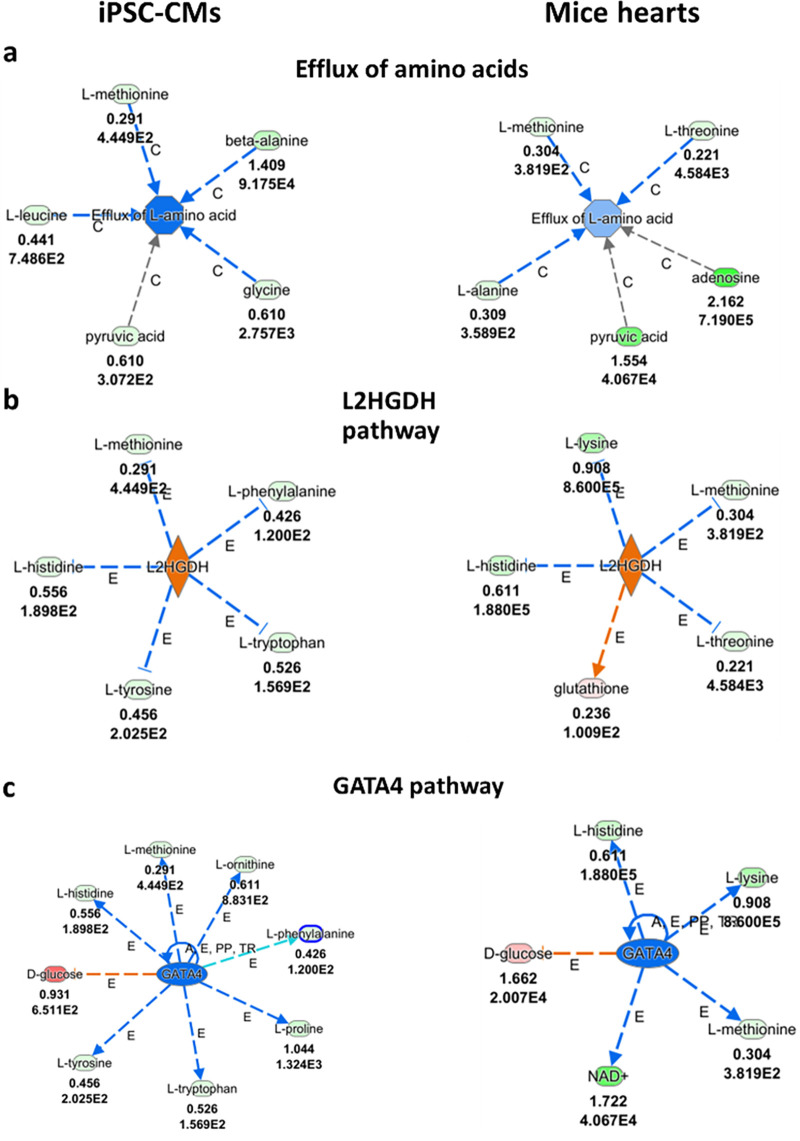
Metabolic pathways related to amino acid metabolism in *PRKAG2*-mutant iPSC-CMs (CMs) and mice hearts, as analyzed by ingenuity pathway analysis (IPA). (**a**) Altered efflux of amino acids in mutant CMs and mice hearts is indicated by higher levels of L-methionine, L-leucine, and beta-alanine (red), suggesting disruptions in amino acid transport. (**b**) Disruptions in the L2HGDH (L-2-hydroxyglutarate dehydrogenase) pathway are observed, with elevated levels of L-phenylalanine, L-histidine, and glutathione (red). These changes suggest enhanced amino acid degradation and shifts in energy metabolism. (**c**) Alterations in the GATA4 pathway are shown, with higher levels of D-glucose, L-methionine, and betaine (red), indicating the pathway's role in regulating amino acid metabolism and the cardiac stress response. The upper numbers refer to log2(fold change); the lower numbers represent the FDR. Color codes: Red = higher levels of metabolites; Green = lower levels of metabolites; Orange = predicted increased function; Blue = predicted decreased function. The curved arrows represent feedback loops or alternative pathway fluxes, while the gradient blue shades correspond to varying levels of predicted inhibition, with darker shades indicating stronger effects.

**Figure 7:**
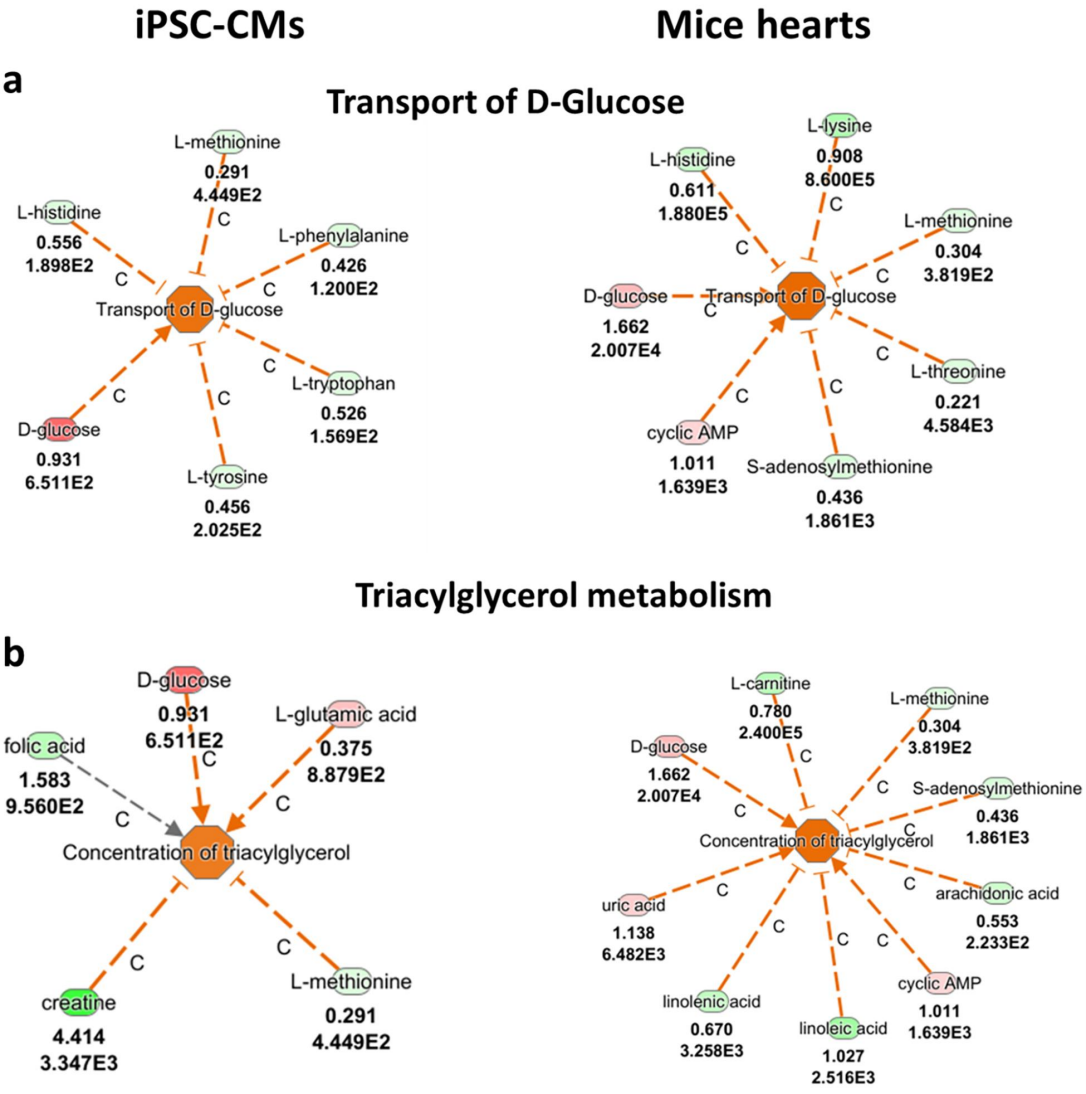
Metabolic pathways related to glucose and lipid metabolism in *PRKAG2*-mutant iPSC-CMs (CMs) and mice hearts, as analyzed by the ingenuity pathway analysis (IPA). (**a**) Altered glucose transport in mutant CMs and mice hearts is indicated by higher levels of D-glucose, L-histidine, and L-methionine (red), suggesting disruptions in glucose handling and energy homeostasis. (**b**) Changes in triacylglycerol (TAG) metabolism are observed, with elevated levels of D-glucose, L-glutamic acid, and L-carnitine (red), indicating increased lipid storage and metabolic dysfunction in both *PRKAG2*-mutant models. The upper numbers refer to log2(fold change), and the lower numbers represent the FDR. Color codes: Red = higher levels of metabolites; Green = lower levels of metabolites; Orange = predicted increased function; Blue = predicted decreased function.

To further explore the metabolic alterations caused by the *PRKAG2* mutation, we performed IPA analysis on the metabolomic data from *PRKAG2*-mutant CMs and mice hearts. As described below, this analysis revealed significant disruptions across multiple pathways, including those related to stress response, amino acid metabolism and energy regulation. To further investigate the metabolic alterations associated with the *PRKAG2* mutation, we analyzed metabolomic data obtained from *PRKAG2*-mutaned CMs and mice hearts. Figures 5–7 summarize the findings, integrating experimental results with computational predictions to provide a comprehensive view of the metabolic disruptions. Figure 5 highlights the primary metabolite changes observed, focusing on pathways related to stress response, amino acid metabolism, and energy regulation. Figures 6, 7 build upon this by incorporating pathway enrichment and predictive modeling to infer upstream regulatory alterations and potential downstream consequences. This prediction process involved mapping metabolite changes to metabolic networks and regulators, offering insights into the broader metabolic impacts of the *PRKAG2* mutation. These figures were generated using Ingenuity Pathway Analysis (IPA), which integrates experimental data with curated biological knowledge to identify disrupted pathways and regulatory mechanisms. Together, these figures bridge raw metabolomic data with biological interpretation, providing valuable insights into the metabolic disruptions driven by the *PRKAG2* mutation.

#### Stress response and energy regulation

Figure 5a shows that *PRKAG2*-mutant CMs have higher levels (red) of metabolites such as D-glucose, L-glutamic acid, and glutathione, suggesting a heightened stress response and increased O-GlcNAc transferase (OGT) activity. Similarly, in *PRKAG2*-mutant mice hearts, Figure 5 highlights corresponding changes in these key metabolites, reflecting a comparable increase in OGT activity. Figure 5b highlights an increase in ROS formation, with higher levels of metabolites like L-carnitine and L-methionine in mutant CMs and mice hearts. The orange connections predict increased oxidative stress and ROS formation in both models. In Figure 5c, the changes in the mTOR pathway are depicted. The orange connections suggest predicted increased mTOR activity, with elevated These findings indicate increased energy regulation and biosynthetic processes in both *PRKAG2*-mutant CMs and mice hearts.

### Amino acid metabolism

Figure 6a shows higher levels of amino acids like L-methionine and beta-alanine in mutant CMs and mouse hearts, indicating a disruption in the efflux of amino acids. These findings suggest impaired amino acid transport, with the green labels indicating lower levels of certain amino acids, such as glycine, contributing to the disruption. Figure 6b shows changes in the L-2-hydroxyglutarate dehydrogenase (*L2HGDH*) pathway, which plays a key role in the metabolism of hydroxyglutarate. Elevated levels (red) of key metabolites, such as L-phenylalanine and L-histidine, alongside the predicted increased activity of *L2HGDH* (orange), suggest enhanced catabolism of hydroxyglutarate in mutant CMs. This enzyme is involved in the detoxification of L-2-hydroxyglutarate, and its upregulation may indicate a compensatory response to altered metabolic flux in the mutant cardiomyocytes. This suggests enhanced amino acid degradation and energy metabolism, especially in the context of *PRKAG2*-mutant models. In Figure 6c, the higher levels of metabolites such as D-glucose and L-methionine suggest increased GATA4 pathway activity, as indicated by the orange predictions of increased function. This implies GATA4's involvement in regulating amino acid metabolism and stress response in both CMs and mice hearts.

### Glucose and lipid metabolism

Figure 7 highlights key metabolic disruptions observed in *PRKAG2*-mutant CMs and mouse hearts. Figure 7a shows elevated levels of D-glucose and related metabolites, suggesting impaired glucose transport and increased metabolic stress. The predicted functional increase (orange) indicates enhanced glucose handling, consistent with dysregulated energy metabolism. Figure 7b reveals elevated levels of L-glutamic acid, L-carnitine, and D-glucose, pointing to heightened triacylglycerol (TAG) metabolism. The predicted increase in lipid storage (orange) reflects metabolic dysfunction and a broad energy storage imbalance in both CMs and mice hearts. These findings underscore the systemic metabolic perturbations associated with the *PRKAG2* mutation. The metabolic profiling data revealed significant disruptions in pathways related to glucose transport and lipid metabolism, including increased triacylglycerol (TAG) accumulation and altered glucose handling (Figure 7). These findings indicate a prominent metabolic imbalance with potential implications for energy storage, lipid accumulation, and mitochondrial function in mutant CMs.

### Lipidomic profiling of *PRKAG2*-mutant iPSC-CMs

**Figure 8:**
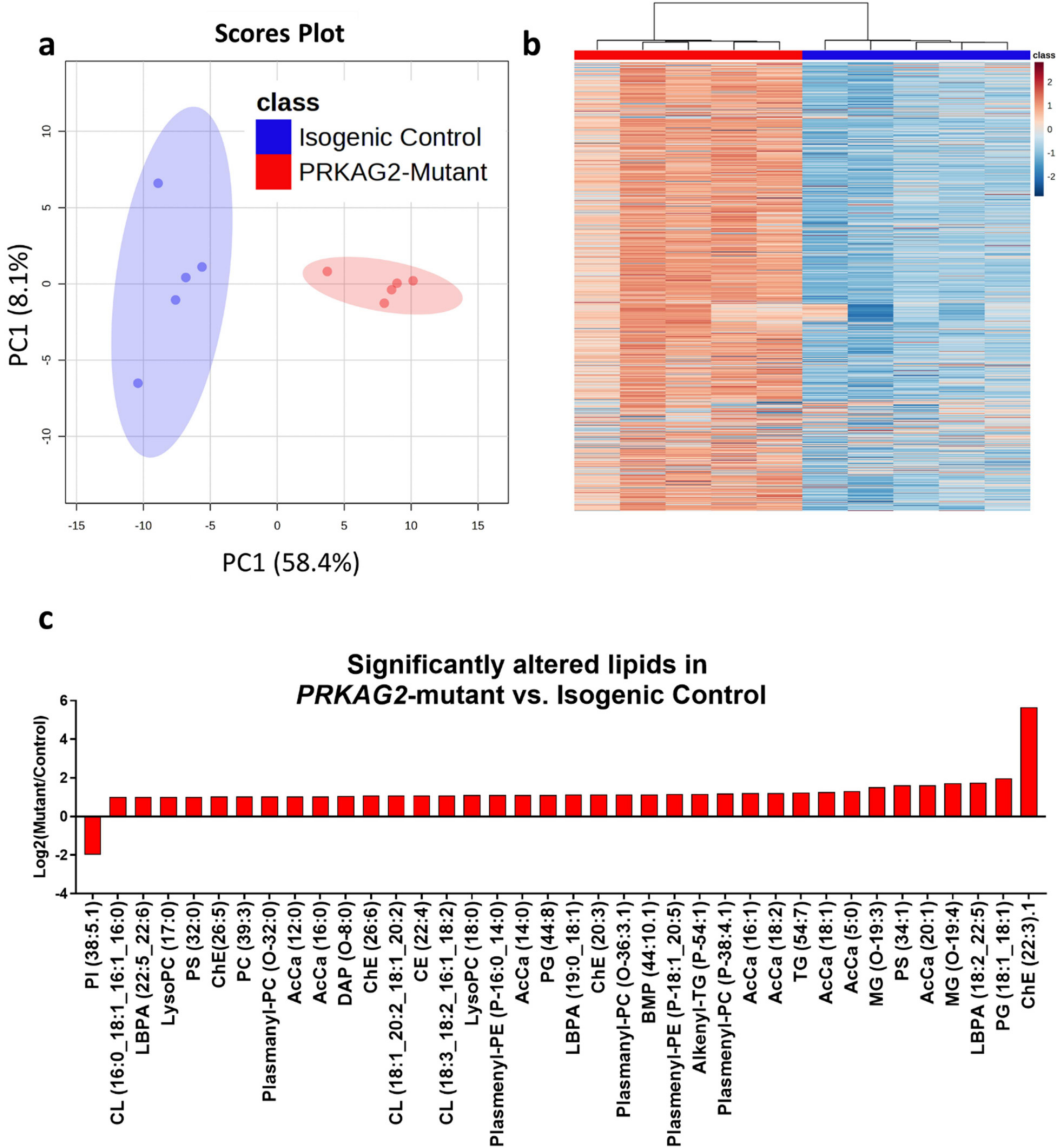
Lipidomic analysis in *PRKAG2*-mutant and isogenic control iPSC-CMs (CMs). (**a**) Scores plot from PCA showing separation between *PRKAG2*-mutant (red, *n* = 5) and isogenic control (blue, *n* = 5) CMs, based on lipidomic data. The PCA demonstrates distinct lipid profiles between the two cell groups, with PC1 accounting for 58.4% of the variation and PC2 accounting for 8.1%. (**b**) Heatmap representing the lipidomic profiles of all 1,051 lipid species detected in *PRKAG2*-mutant and isogenic control CMs. Lipid abundance is represented by a color gradient, with red indicating higher values and blue indicating lower values. The heatmap shows clear clustering of the samples, highlighting the differences in lipid profiles between the two groups. (**c**) A bar plot showing fold changes of lipid species with at least a 2-fold difference between mutant and isogenic control CMs. Positive and negative fold changes represent both upregulation and downregulation of lipid species in the mutant cells relative to controls.

To investigate further the lipid alterations identified in the metabolomic analysis and to understand the role of disrupted lipid metabolism in the observed metabolic dysfunction caused by the *PRKAG2* mutation, we conducted a comprehensive lipidomics study on mutant CMs. This analysis included over 1,000 lipid species, particularly TAGs and other key lipids, focusing on those showing at least a 2-fold change between *PRKAG2*-mutant and isogenic control CMs. The lipidomics data provided additional insight into the disrupted lipid metabolism previously observed in metabolomics and energy regulation studies. Figure 8 shows the distinct lipid profiles in *PRKAG2*-mutant and isogenic control CMs, as reflected by the PCA and the overall distribution of lipid species. In Figure 8a, the PCA shows a clear separation between *PRKAG2*-mutant and isogenic control CMs, with PC1 accounting for 58.4% of the variance, indicating significant differences in lipid composition between the two groups. The clustering of samples in the PCA aligns with the observed metabolic disturbances, supporting the notion of distinct lipidomic signatures in the mutant CMs. Figure 8b presents a heatmap of all 1,051 lipid species, showing the global lipidomic differences between *PRKAG2*-mutant and isogenic control CMs. Lipid abundance is represented by a color gradient, with red indicating higher abundance and blue indicating lower abundance. This heatmap reveals clear clustering of mutant and CRISPR-corrected samples, suggesting that lipid metabolism is broadly affected by the *PRKAG2* mutation, resulting in distinct lipid profiles in the mutant CMs. Figure 8c illustrates log2 (fold changes) of lipid species, showing at least a 2-fold difference between *PRKAG2*-mutant and isogenic control CMs. This bar plot captures both upregulated and downregulated lipid species in the mutant CMs, reflecting the dynamic nature of lipid metabolism in CMs model. The significant changes observed in several lipid species suggest an overall imbalance in lipid homeostasis in mutant CMs.

#### Lipid storage and accumulation

Consistent with the findings from the metabolomic analysis (Figure 7), the lipidomic profiling reveals a significant upregulation of lipid species associated with storage, particularly cholesterol esters (ChE). These lipid classes showed fold changes such as ChE (22:3) (5.67), ChE (26:5) (1.03), and ChE (26:6) (1.08), indicating an accumulation of neutral lipids and disruptions in lipid storage processes. Additionally, monoacylglycerol (MG) species were also elevated, including MG (O-19:3) (1.52) and MG (O-19:4) (1.71). These increases may reflect heightened triacylglycerol (TAG) turnover in *PRKAG2*-mutant CMs, with enhanced lipolysis (breakdown of TAG into MG and fatty acids) alongside potential disturbances in lipid utilization or storage capacity.

#### Energy metabolism and acyl carnitines (AcCa)

The lipidomic analysis revealed a 2.36-fold upregulation of acyl carnitine (AcCa) species, which are key intermediates in fatty acid oxidation. Acyl carnitines facilitate the transport of fatty acids into the mitochondria for beta-oxidation, and their accumulation in mutant CMs suggests dysregulation in fatty acid metabolism. This increase in AcCa species aligns with the hypothesis of altered mitochondrial fatty acid utilization in mutant CMs, consistent with previously observed changes in mitochondrial respiration.

#### Phospholipid metabolism and membrane integrity

Significant changes were observed in phosphatidylserine (PS) species, such as PS (34:1) (1.62) and PS (32:0) (1.02), as well as in phosphatidylglycerol (PG) species, including PG (44:8) (1.12) and PG (18:1_18:1) (1.97). These results indicate alterations in phospholipid metabolism, which are critical for maintaining mitochondrial structure and function. Phosphatidylglycerol is a precursor of cardiolipin, a phospholipid essential for mitochondrial membrane integrity and optimal respiratory chain activity. The observed changes in PS and PG species suggest altered phospholipid composition, consistent with the metabolic dysfunction identified in *PRKAG2*-mutant CMs.

### RNA-seq analysis

**Figure 9:**
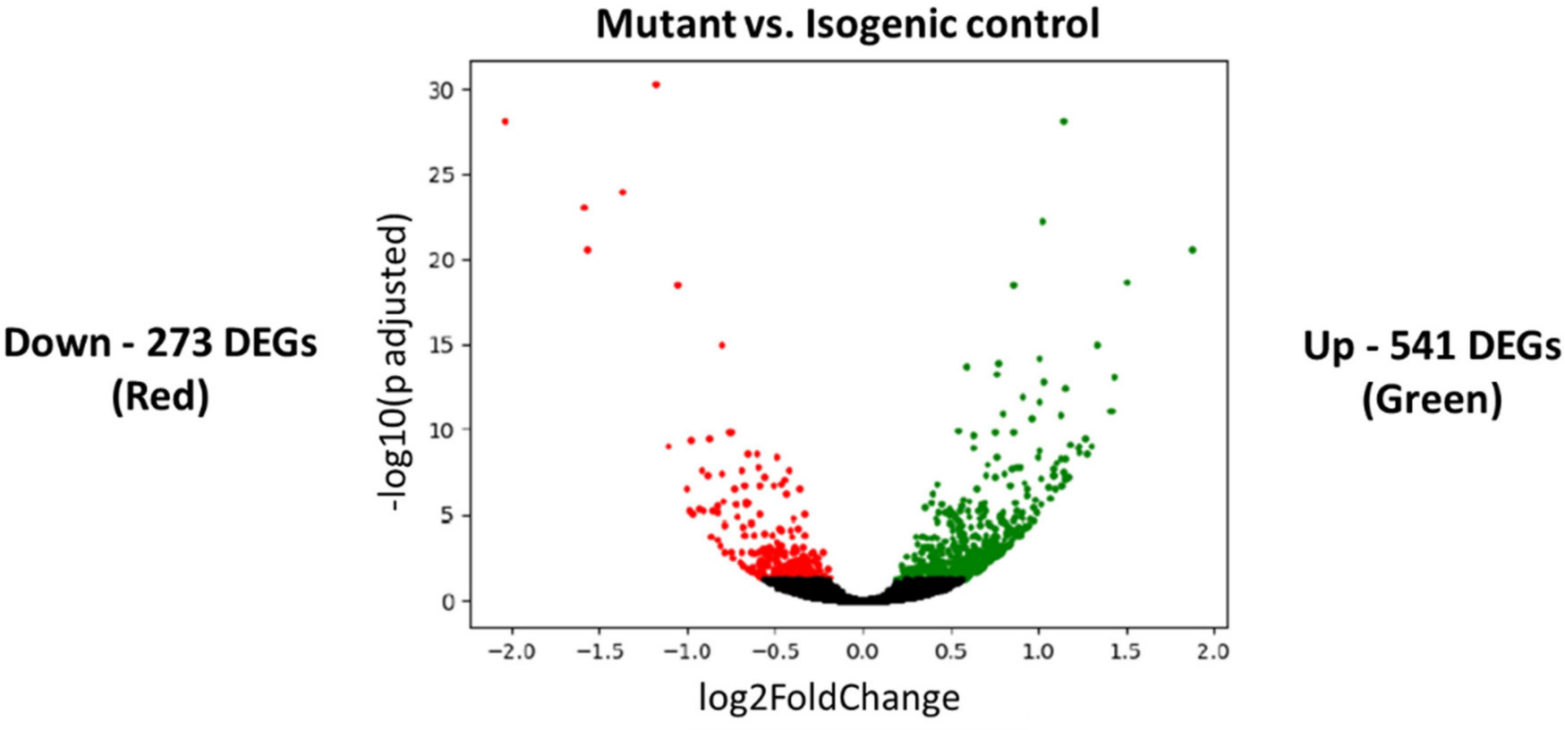
Differentially expressed genes (DEGs) in PRKAG2-mutant vs. isogenic control iPSC-CMs (CMs). Volcano plot representing differentially expressed genes (DEGs) in mutant CMs compared to isogenic control CMs. Each dot represents a single gene, with green dots representing upregulated genes (541 DEGs) and red dots representing downregulated genes (273 DEGs). The *x*-axis shows the log2 fold gene expression change (mutant vs. isogenic control CMs), while the *y*-axis represents the −log10 of the adjusted *p*-value, indicating the statistical significance of gene expression changes. Genes with *p*-adjusted < 0.05 are considered significantly differentially expressed.

To investigate the gene expression changes between mutant and isogenic control CMs, we performed RNA-seq analysis. This analysis provided a view of the transcriptional differences associated with the mutation, highlighting key metabolic and regulatory pathways that are disrupted in mutant CMs. As shown in Figure 9, the RNA-seq analysis identified a total of 814 differentially expressed genes (DEGs) between mutant and isogenic control CMs, with 541 genes upregulated (green) and 273 genes downregulated (red) in the mutant CMs. The volcano plot in Figure 9 illustrates the distribution of these DEGs, with significant changes in gene expression denoted by high −log10(*p* adjusted) values. These findings suggest substantial transcriptional alterations due to the *PRKAG2* mutation.

### Key pathways affected by the *PRKAG2^R302Q^* mutation

Table 1 summarizes the RNA-seq results, categorizing DEGs into relevant pathways to assess the impact of the mutation on cellular processes. The analysis reveals significant changes in pathways related to glycogen synthesis, glycolysis, mitochondrial function, lipid metabolism, oxidative stress and cardiac hypertrophy signaling.

**Table 1:** RNA-seq analysis of differentially expressed genes (DEGs) in PRKAG2-mutant vs. isogenic control iPSC-CMs.

Pathway	Key genes	Up/downregulated genes	Overall pathway regulation
Glycogen synthesis	IGFBP3, IRS2, PGM1	IGFBP3 ↑, IRS2 ↑↑, PGM1 ↑	Upregulated
Glycolysis	GCK, PFKFB3, PKM, LDHA	GCK ↑, PFKFB3 ↓, PKM ↑, LDHA ↑	Mixed regulation
Oxidative stress	NOX4, HMOX1, GPX3	NOX4 ↑, HMOX1 ↑, GPX3 ↑	Upregulated
Mitochondrial function	OPA1, TXN2, SAMM50	OPA1 ↑, TXN2 ↑, SAMM50 ↑	Upregulated
Lipid metabolism	ACADVL, PPARA	ACADVL ↑, PPARA ↑	Upregulated
Cardiac hypertrophy	PRKAG2, MYH7, ACTC1, IGF1, GATA4, TNNT2	PRKAG2 ↑, MYH7 ↑, ACTC1 ↑, IGF1 ↑, GATA4 ↑, TNNT2 ↑	Upregulated

#### Glycogen synthesis

RNA-seq analysis confirmed significant upregulation of key genes involved in glycogen synthesis, including *IGFBP3*, *IRS2*, and *PGM1* (Table 1). These findings align with the observed glycogen accumulation in the mutant CMs, suggesting a shift towards enhanced glycogen storage as a result of the *PRKAG2* mutation. Notably, *IGFBP3* was upregulated by 0.432 log2 fold change (Isogenic control vs. Mutant CMs), which plays a role in regulating insulin-like growth factor signaling and promotes glycogen synthesis. This suggests that glycogen storage is an integral part of the metabolic imbalance in *PRKAG2* cardiomyopathy.

#### Glycolysis

Despite increased glycogen synthesis, a mixed regulatory pattern was observed in the glycolytic pathway. Genes such as *GCK* (glucokinase) and *PKM* (pyruvate kinase) showed slight upregulation, with log2 fold changes of 0.005 and 0.112, respectively. In contrast, *PFKFB3*, a key regulator of glycolysis, was downregulated by −0.123. The downregulation of *PFKFB3* aligns with the observed reduction in glycolytic capacity in mutant CMs, as demonstrated by Seahorse analysis (Figure 1). Previous studies have reported increased glucose uptake in *PRKAG2*-mutant hearts (27), which may contribute to the observed metabolic alterations. These findings suggest that while some glycolytic genes are upregulated, overall glycolytic function is likely reduced, reflecting impaired metabolic regulation in mutant CMs.

#### Mitochondrial function and oxidative stress

Several genes associated with mitochondrial function were significantly upregulated in mutant CMs, consistent with increased mitochondrial activity. Notable genes include *OPA1* (0.028 log2 fold change), involved in mitochondrial dynamics and fusion, *TXN2* (0.136 log2 fold change), which regulates mitochondrial redox balance, and *SAMM50* (0.044 log2 fold change), which is critical for maintaining mitochondrial membrane integrity. These findings align with the increased maximal respiration observed in mutant CMs (Figure 2), suggesting enhanced mitochondrial biogenesis and activity. In parallel, oxidative stress-related genes such as *NOX4* (0.317 log2 fold change), *HMOX1* (0.098 log2 fold change), and *GPX3* (0.081 log2 fold change) were also upregulated. These genes are involved in reactive oxygen species (ROS) production and detoxification, indicating elevated oxidative stress in mutant CMs. This upregulation corroborates the metabolomics data showing increased ROS formation in mutant cells (Figure 5), further highlighting the link between mitochondrial overactivity and oxidative stress in mutant CMs.

#### Lipid metabolism

RNA-seq analysis showed that lipid metabolism is significantly activated in mutant CMs. Genes involved in fatty acid oxidation, such as *ACADVL* (0.025 log2 fold change), and lipid metabolism regulation, such as *PPARA* (0.056 log2 fold change), were upregulated. These results support the lipidomics findings (Figure 8), which show an increased accumulation of triacylglycerols (TAGs) and other lipid species. The upregulation of lipid metabolism genes likely reflects a shift towards increased lipid oxidation and storage, compensating for impaired glycolytic function.

### Cardiac hypertrophy signaling

Cardiac hypertrophy signaling is significantly upregulated in mutant CMs (28). Key genes, including *PRKAG2* itself [0.564 log2 (fold change)], *ANKRD1* [0.37 log2 (fold change)], *MYH7* [0.311 log2 (fold change)] and *IGF1* [0.421 log2 (fold change)] are upregulated, indicating enhanced hypertrophic signaling. *GATA4*, a critical transcription factor involved in cardiac hypertrophy, is upregulated by 0.193 log2 (fold change), further supporting the link between metabolic dysregulation and hypertrophic remodeling in *PRKAG2* cardiomyopathy. These findings suggest that the metabolic alterations in the mutant CMs drive hypertrophic signaling pathways, contributing to the disease phenotype.

### The effect of metformin on mitochondrial activity in *PRKAG2*-mutant CMs

**Figure 10:**
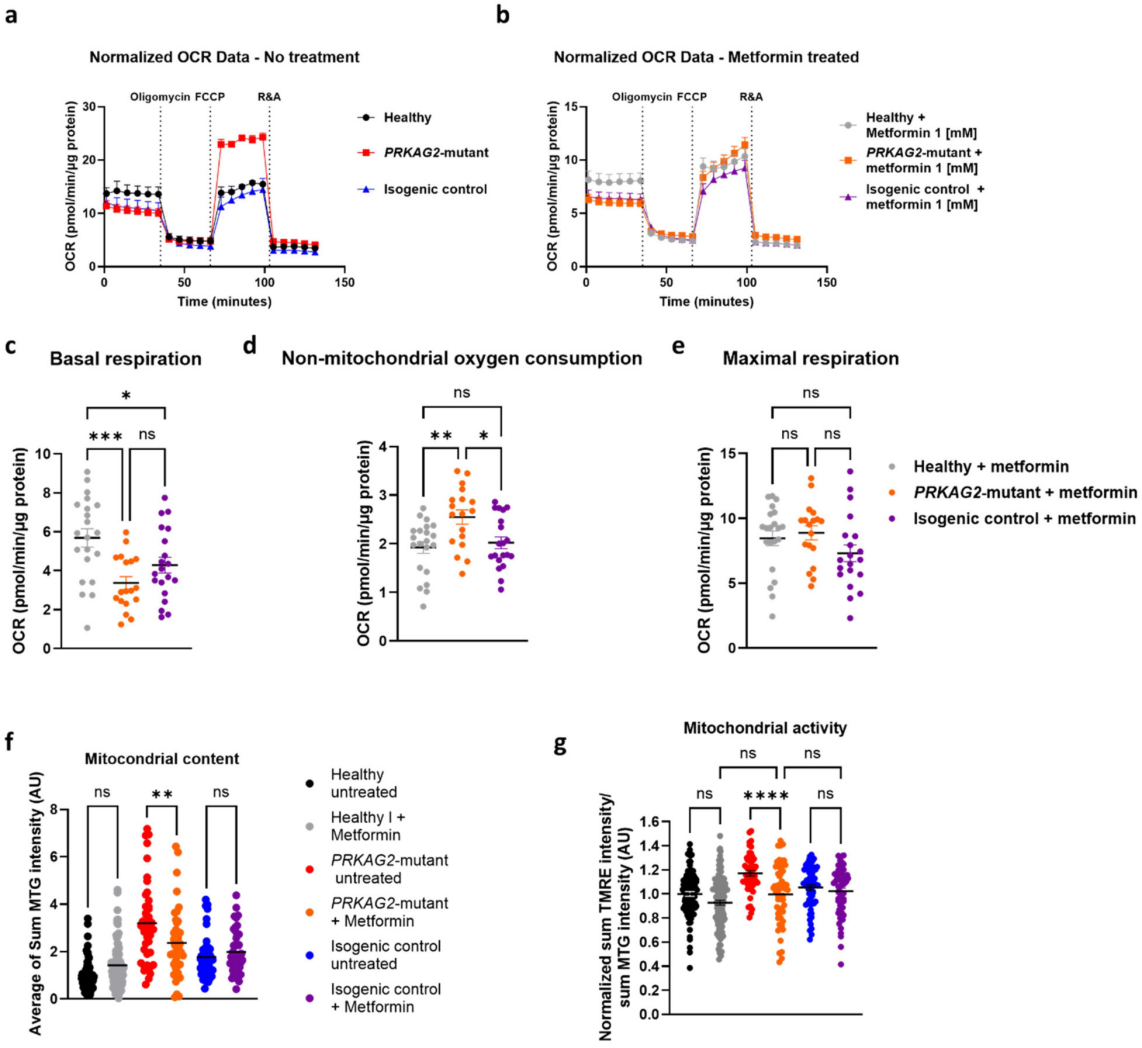
The effect of metformin treatment on respiration and mitochondria. (**a,b**) Oxygen consumption rate (OCR) in (**a**) untreated *PRKAG2*-mutant (red), isogenic control (blue) and healthy (black) CMs, and (**b**) mutant (deep red), isogenic control (dark blue) and healthy (grey) CMs treated with 1 mM metformin for 24 h. (**c–e**) Basal respiration, non-mitochondrial oxygen consumption, and maximal respiration rates are calculated based on plot b. (**f**) Quantification of total mitochondrial content by MTG intensity. (**g**) Quantification of total mitochondrial activity calculated by the ratio of TMRE over MTG fluorescence (TMRE/MTG), of each image. The summary demonstrates that 24-hour metformin treatment decreased maximal respiration in the mutant CMs to the level of isogenic control and healthy CMs, while having no effect on non-mitochondrial OCR. In addition, metformin treatment caused a significant decrease in mitochondrial content and activity in mutant CMs, compared to isogenic control and healthy CMs. The statistical significance is as follows: (**a**–**e**) Healthy, *n* = 21; *PRKAG2*-mutant, *n* = 18; isogenic control, *n* = 20. The Shapiro–Wilk test for normality was performed on the data. One-way ANOVA was followed by the Tukey multiple comparisons test.; NS = non-significant, *p < 0.05, **p < 0.01, ***p < 0.001. (**f,g**) Healthy, *n* = 94; Mutant, *n* = 57; Isogenic control, *n* = 59. One-way ANOVA, ns = non-significant, *p < 0.05, **** *p* < 0.0001.

Given our findings of increased mitochondrial activity in mutant CMs, we hypothesized that treatment with the AMPK activator metformin (1 mM) would reduce mitochondrial activity and respiration in mutant CMs, without affecting healthy CMs. Metformin is known to inhibit mitochondrial function at supra-pharmacological concentrations (13, 15, 16). To test the hypothesis, a Mito Stress Test was conducted in both untreated (Figure 10a) and mutant CMs treated with metformin for 24 h (Figure 10b). Untreated mutant CMs exhibit a significantly increased oxygen consumption rate (OCR) after FCCP injection, compared to isogenic control and healthy CMs, consistent with the findings shown in Figure 2. After 24 h of metformin treatment, basal respiration was reduced in both mutant and isogenic control CMs, compared to healthy CMs (Figure 10c). Furthermore, metformin treatment did not affect non-mitochondrial oxygen consumption (Figure 10d), with mutant CMs displaying a ∼50% increase in non-mitochondrial oxygen consumption compared to isogenic control and healthy CMs, aligning with the results observed in untreated CMs (Figure 2c). Notably, following metformin treatment, maximal respiration was similar across all three groups (Figure 10e), indicating a normalization of mitochondrial respiration in mutant CMs. Next, we measured the mitochondrial content (Figure 10f) and activity (Figure 10g) in metformin-treated CMs. In mutant CMs, metformin caused a 25% decrease in mitochondrial content and a 20% decrease in mitochondrial activity, whereas isogenic control and healthy CMs were unaffected by metformin. These results suggest that metformin selectively reduces mitochondrial content and activity in mutant CMs while leaving isogenic control and healthy CMs unaffected, highlighting its potential therapeutic specificity for addressing mitochondrial dysfunction in the context of *PRKAG2* cardiomyopathy.

### Metformin blocks the arrhythmias in *PRKAG2*-mutant iPSC-CMs

**Figure 11:**
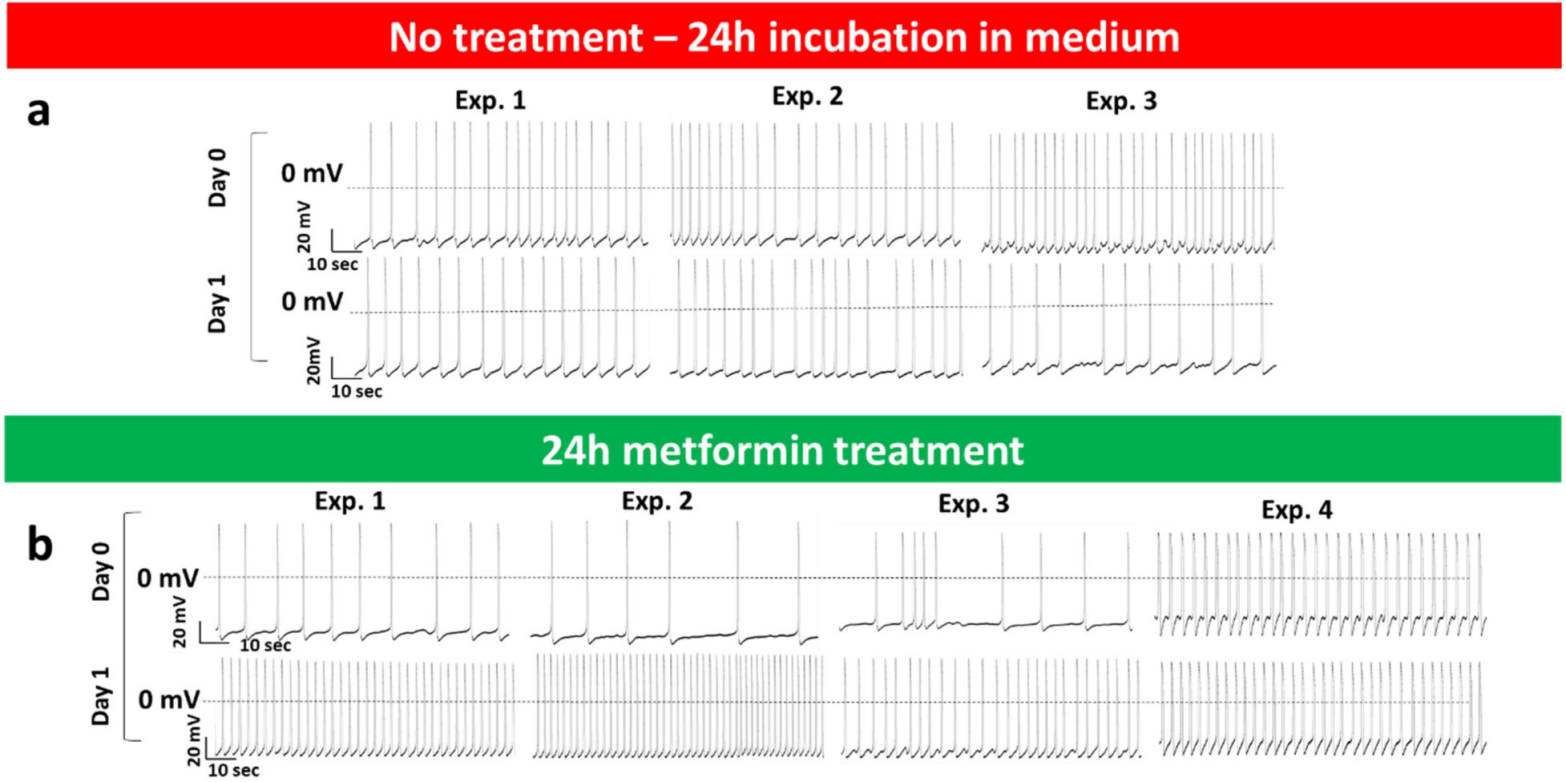
Metformin blocks the arrhythmias in *PRKAG2*-mutant iPSC-CMs. (**a**) Action potentials of untreated mutant CMs at Day 0 (upper row) and after 24-hour (Day 1) incubation in fresh medium (lower row). As shown, arrhythmogenic activity persisted at both time points. (**b**) Action potentials of mutant CMs at Day 0 (upper row) and 24 h (Day 1) after treatment with 1 mM metformin (lower row). In Experiments 1, 2 and 4 metformin treatment prevented the arrhythmias, whereas in Experiment 3, the arrhythmogenic phenotype was markedly attenuated.

**Figure 12:**
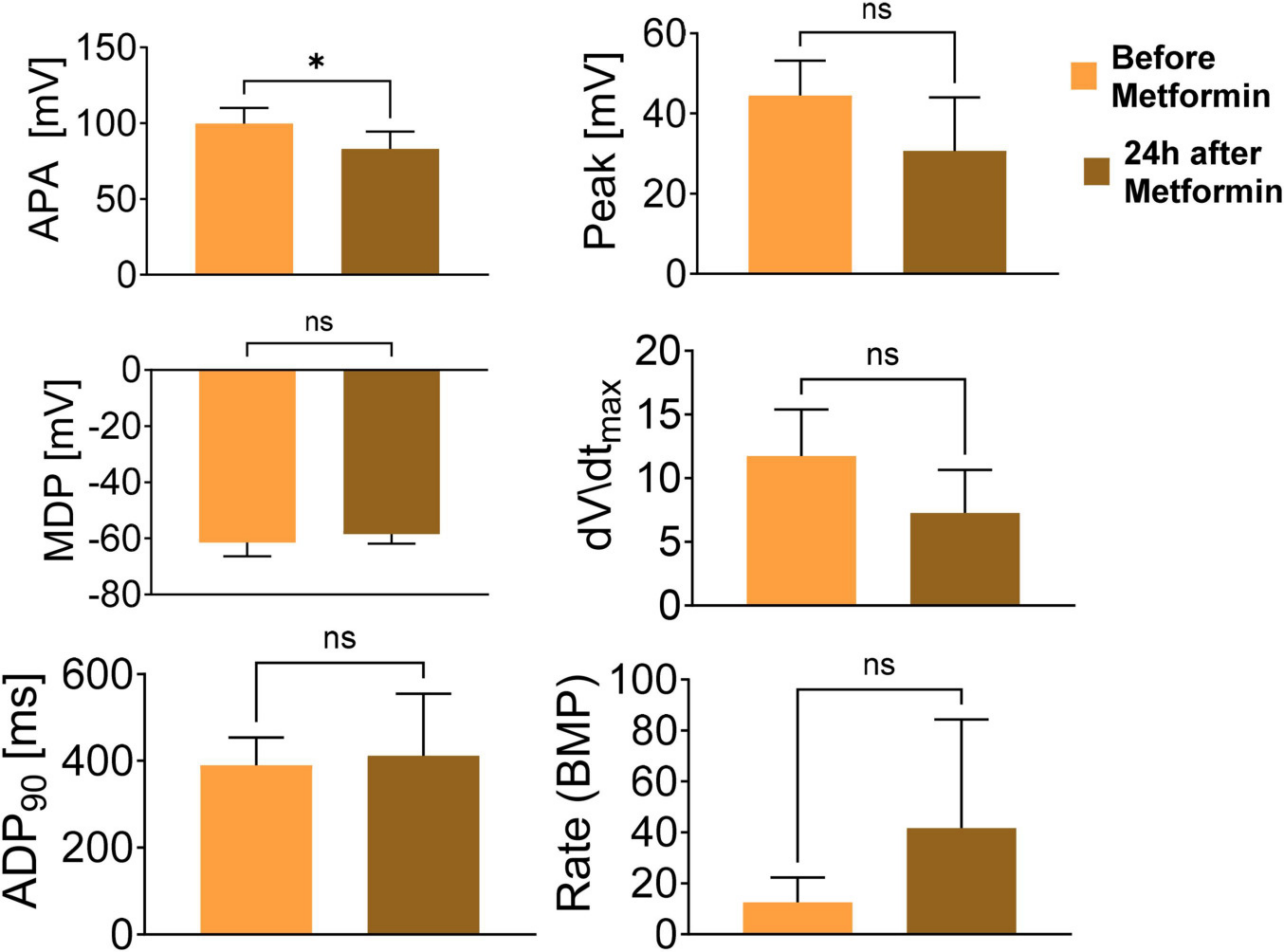
Action potential characteristics of *PRKAG2*-mutant iPSC-CMs (CMs) in the absence and presence of metformin. Characteristics of the action potentials shown in Figure 11b at day 0 and after 24-hour (Day 1) incubation with metformin. APA: action potential amplitude; Peak: action potential peak; MDP: maximal diastolic potential; dV/dt_max_: maximal upstroke velocity of phase zero depolarization; APD_90_: action potential duration at 90% repolarization; Rate: Beats per minute. Comparisons between the untreated and treated CMs was performed by means of unpaired t-test. *p < 0.05. *n* = 6 CMs in each group.

As we reported earlier (7), mutant CMs present prominent arrhythmias, suggesting a direct electrophysiological impairment caused by the *PRKAG2*^R302Q^ mutation. To test whether metformin treatment can alleviate the arrhythmias, we recorded action potentials (AP) from mutant CMs before and after metformin treatment. As shown in Figure 11a (upper row), action potential measurements of untreated mutant CMs illustrate the presence of arrhythmias, indicated by irregular spontaneous firing patterns and sub-threshold oscillatory potentials. The arrhythmias persisted on Day 0 and after 24-hour incubation in fresh medium (Day 1). The upper row in Panel b shows arrhythmogenic action potentials recorded from mutant CMs on Day 0. As shown in the lower row, following 24-hour incubation in 1 mM metformin (Day 1), in Experiments 1, 2 and 4 metformin treatment prevented the arrhythmias, whereas in Experiment 3, the arrhythmogenic phenotype was markedly attenuated. These findings indicate that metformin can reduce or eliminate electrophysiological abnormalities in mutant CMs. As shown in Figure 12, except for a small decrease in action potential amplitude (APA), 24-hr incubation with metformin, it blocked the arrhythmias (Figure 11) without affecting action potential characteristics. As shown in Figure 12, except for a small decrease in action potential amplitude (APA), 24-hr incubation with metformin, it blocked the arrhythmias (Figure 11) without affecting action potential characteristics.

## Discussion

In the present study we tested the hypothesis that the *PRKAG2* mutations R302Q and T400N lead to bioenergetic, metabolic and electrophysiological alterations in cardiomyocytes, contributing to the pathophysiology of WPW syndrome. The major findings are: (1) *PRKAG2*-mutant CMs and mutated murine hearts exhibit disrupted glycogen metabolism, including glycogen accumulation and reduced glycolytic flux; (2) *PRKAG2*-mutant models demonstrate mitochondrial remodeling characterized by increased mitochondrial content and elevated maximal respiratory capacity while basal oxidative phosphorylation remains unchanged; (3) lipid metabolism is altered, with increased reliance on fatty acid oxidation and accumulation of lipid species; (4) metformin treatment attenuated arrhythmic activity and modulated mitochondrial parameters in mutant CMs.

### Glycogen accumulation and glycolytic dysregulation in *PRKAG2*-mutant CMs

A hallmark of *PRKAG2* cardiomyopathy is glycogen accumulation. Our findings demonstrate upregulated glycogen synthesis pathways in mutant CMs, consistent with enhanced glycogen deposition observed in previous studies (7, 29, 30). Specifically, the observed upregulation of genes such as *IGFBP3*, *IRS2* and *PGM1*, mirrors the transcriptional changes identified in the mutant models, suggesting a conserved mechanism across studies. Additionally, the RNA-seq data highlight downregulation of *PFKFB3* and upregulation of *PKM* and *LDHA*, suggesting a metabolic bottlenecks, similar to those reported by Zhan et al. (30), where glycolysis was reduced despite increased glycogen storage. This paradoxical shift underscores a metabolic rerouting caused by *PRKAG2* mutations, as previously described (29).

Indicated by the results from the metabolic analysis, the novel finding of increased OGT activity in mutant CMs likely plays a role in this metabolic dysregulation. OGT, responsible for glycosylating proteins in response to glucose flux, may further influence glucose metabolism by modulating protein function in glycolysis and glycogen synthesis pathways. Prior studies showed that OGT activity is linked to various metabolic disorders (31), and hence our data suggests that it may contribute to the metabolic imbalances in *PRKAG2* cardiomyopathy. This finding warrants further exploration, as targeting OGT could represent a new therapeutic approach in addressing metabolic dysregulation in *PRKAG2* mutations.

### Mitochondrial function and increased oxidative stress in *PRKAG2*-mutant CMs

The mitochondrial alterations observed in mutant CMs aligns with increased maximal respiratory capacity reported in both human and murine models (29, 30). The Seahorse Mito Stress results show that mutant CMs have significantly higher maximal respiration and spare respiratory capacity than isogenic control and healthy CMs, while basal respiration remains comparable between groups. This indicates an expansion of the respiratory reserve rather than an increase in basal oxidative phosphorylation. The increased maximal respiratory capacity aligns with the observed upregulation of genes associated with mitochondrial biogenesis, including *OPA1* involved in mitochondrial fusion; *PPARGC1A*, a key regulator of mitochondrial biogenesis; and *NDUFA4*, a part of complex I in the electron transport chain. Together with the increased mitochondrial content observed in mutant CMs, these findings suggest mitochondrial remodeling rather than constitutive mitochondrial overactivation. Both metabolomic and RNA-seq analyses identified changes in pathways associated with redox regulation, specifically, upregulation of *NOX4* and *HMOX1*. While these findings are consistent with altered redox homeostasis, increased ROS production cannot be definitively concluded since direct measurements of intracellular ROS levels were not performed in the present study. Previous studies have linked mitochondrial remodeling and redox imbalance to metabolic cardiomyopathies (32); accordingly, modulation of redox pathways may represent a potential therapeutic avenue for *PRKAG2* cardiomyopathy. While metformin was associated with modulation of mitochondrial parameters, the role of direct ROS scavengers in *PRKAG2*-mutant CMs remains to be determined. Studies other metabolic cardiomyopathy models have shown promising results with mitochondria-targeted antioxidants. In Barth syndrome iPSC-CMs, treatment with mitoTEMPO effectively reduced ROS levels, improved Ca^2+^ handling, and decreased arrhythmogenic Ca^2+^ release events (33). Similarly, in Danon disease iPSC-CMs, treatment with N-acetylcysteine (NAC) lowered oxidative stress and improved mitochondrial morphology and function (34). Additionally, NAC prevented arrhythmia-induced Ca^2+^ handling deterioration in a simulated atrial fibrillation iPSC-CMs model, demonstrating its protective effects on electrophysiological stability (35). Although ROS scavengers have not been tested in *PRKAG2*-mutant iPSC-CMs, these findings from related metabolic cardiomyopathy models support further investigation into their potential therapeutic relevance.

### The correlation between mice and CM metabolomics

To the best of our knowledge, this is the first study which performs a comprehensive metabolomics profiling of *PRKAG2*-mutant murine hearts and compares the results with human mutant CM data. The metabolomic analysis of the murine heart tissue showed a significant accumulation of glycolytic intermediates alongside alterations in tricarboxylic acid (TCA) cycle activity, findings that mirror those observed in the mutant CMs. In both models, there was a shift in the metabolic balance with increased lipid storage and changes in mitochondrial parameters. Specifically, the increased levels of acyl-carnitines in both the murine and CM models may reflect modifications in fatty acid utilization in the context of the reduced glycolysis observed in these cells (7). The murine data revealed elevated levels of long-chain acyl-carnitines and changes in glycolytic metabolites, indicating a metabolic reorganization associated with the *PRKAG2* mutation. Similarly, CMs displayed increased maximal respiratory capacity and reduced glycolytic flux, further supporting the conserved metabolic phenotype. These findings suggest that *PRKAG2* mutations affect core energy pathways in a manner consistent across species. This cross-species alignment provides robust validation for the use of both murine and CM models in understanding *PRKAG2* cardiomyopathy.

### Lipid metabolism and storage dysregulation in *PRKAG2*-mutant iPSC-CMs

Comprehensive lipidomic analysis uncovered significant alterations in lipid metabolism in mutant CMs. The most striking findings were the increased accumulation of triacylglycerols (TAGs), monoacylglycerol (MG) species and cholesterol esters (ChE). These results suggest that *PRKAG2* mutations lead to an imbalance in lipid storage processes, contributing to the metabolic dysfunction observed in the mutant CMs. The accumulation of neutral lipids, particularly TAGs, aligns with previous studies suggesting that metabolic storage disorders are a hallmark of *PRKAG2*-related cardiomyopathies (36). The upregulation of lipid metabolism genes, including *ACADVL* (involved in fatty acid oxidation) and *PPARA* (a regulator of lipid metabolism), further supports the notion that mutant CMs exhibit a metabolic shift toward lipid storage and oxidation. This may represent a compensatory mechanism in response to impaired glucose metabolism, where cells rely more on lipid storage and oxidation for energy production. However, this shift comes at the cost of increased lipid accumulation, which can disrupt normal cellular function and contribute to the progression of cardiomyopathy. The lipidomic data also reveal a moderate upregulation of acyl carnitines (AcCa), critical intermediates in fatty acid oxidation. Acyl carnitines have a key role in transporting fatty acids into the mitochondria for beta-oxidation, and their accumulation in mutant CMs suggests a dysregulation in fatty acid metabolism. This is consistent with the increased mitochondrial respiration observed in the mutant CMs and suggests that fatty acid oxidation is contributing to the metabolic imbalance in *PRKAG2* cardiomyopathy. Additionally, we observed significant changes in phospholipid metabolism, particularly in phosphatidylserine (PS) and phosphatidylglycerol (PG) species. The marked upregulation of PS (34:1) and PG (44:8) suggests alterations in membrane composition and integrity, which could have far-reaching effects on cellular function, particularly in organelles like mitochondria. Phospholipids play a critical role in maintaining membrane dynamics, and their dysregulation can impair mitochondrial function, exacerbating the bioenergetic defects observed in mutant CMs. Finally, our findings highlight potential disruptions in lipid trafficking and lysosomal function, as evidenced by the upregulation of lysobisphosphatidic acid (LBPA) species, including LBPA (19:0_18:1) and LBPA (18:2_22:5). LBPA is known to have a critical role in endosomal/lysosomal membrane dynamics and lipid transport, and its dysregulation may contribute to the accumulation of lipids within cellular compartments. Impaired lipid trafficking could further exacerbate mitochondrial dysfunction, leading to increased oxidative stress and the progression of cardiomyopathy. These findings are consistent with previous studies that reported an upregulation of lipid metabolism pathways and enhanced fatty acid oxidation in *PRKAG2* models (30). Elevated levels of acyl-carnitines, critical intermediates in fatty acid oxidation, suggest mitochondrial reliance on fatty acid metabolism as a compensatory mechanism, a pattern also observed in the murine models of *PRKAG2* cardiomyopathy (29).

### Modulation of mitochondrial content and function by metformin

An intriguing aspect of this study is the selective effect of metformin on mitochondrial function in mutant CMs. Metformin, often described as an AMPK activator, reduced mitochondrial content by ∼25% and mitochondrial activity by ∼20% in mutant CMs, with no significant effects on the isogenic control or healthy CMs. These findings indicate that metformin modulates mitochondrial properties in mutant CMs and is associated with a partial normalization of respiratory parameters. While metformin is known to activate AMPK, its primary mechanism of action at clinically relevant concentrations is generally attributed to AMPK activation. Although inhibition of complex I has been reported, this effect has been described mainly at supra-pharmacological concentrations (37). Given that *PRKAG2* mutations result in dysregulated AMPK activity, it is plausible that metformin exerts its beneficial effects, at least in part, through modulation of AMPK-dependent pathways rather than through direct mitochondrial inhibition. However, as we did not directly measure ATP levels, further investigation is needed to determine the precise downstream mechanisms involved.

The normalization of basal and maximal respiration in mutant CMs by metformin is consistent with an adjustment of mitochondrial content and respiratory reserve. Notably, the reduction in mitochondrial content was proportionally greater than the reduction in overall respiratory activity, suggesting that the remaining mitochondrial pool may retain or even concentrate functional capacity (38). Importantly, the selective effect of metformin on mutant CMs, while sparing healthy and isogenic control CMs, suggests that metformin could be a promising therapeutic agent for *PRKAG2* cardiomyopathy. The ability of metformin to reduce mitochondrial content and activity without adversely affecting normal cellular function highlights its potential as a targeted therapy for this condition.

### Metformin abolished the arrhythmias in *PRKAG2*-mutant iPSC-CMs

The arrhythmias observed in mutant CMs, represent a key pathological feature of this model. Untreated mutant CMs exhibit prominent arrhythmias, consistent with the clinical presentation of *PRKAG2*-related WPW syndrome, which is characterized by pre-excitation and atrial fibrillation (39). These findings further confirm the intrinsic link between metabolic dysfunction and electrophysiological abnormalities in *PRKAG2* cardiomyopathy. The most compelling result of this study was the significant reduction in arrhythmic events following metformin treatment. After 24 h of exposure to metformin, most arrhythmias in mutant CMs were eliminated, suggesting that metformin not only modulates metabolic parameters but also exerts a stabilizing effect on the electrophysiological properties of the mutant CMs. The mechanism by which metformin alleviates arrhythmias in mutant CMs is likely multifactorial. Given the central role of AMPK in cellular energy sensing and ion channel regulation, activation or modulation of AMPK signaling may contribute to improved electrophysiological stability. AMPK has been shown to influence the activity of Na^+^, K^+^ and Ca^+^ channels, which are critical for maintaining normal cardiac rhythm (40).

Beyond these metabolic effects, metformin can also act directly on ion channels, independently of its impact on mitochondrial function. Metformin has been reported to inhibit L-type Ca^2+^ channel (I_Ca,L_), which may contribute to its antiarrhythmic properties. Whereas metabolic effects may involve adjustments in mitochondrial content and cellular energy balance, electrophysiological effects may also arise from direct modulation of ion channel activity, leading to a rapid stabilization of action potentials. Further research is needed to fully delineate the contributions of these metabolic and electrophysiological mechanisms in the context of *PRKAG2* mutations. Nevertheless, our findings demonstrate that metformin attenuates the arrhythmias in *PRKAG2*-mutant CMs, supporting its potential therapeutic relevance in *PRKAG2*-associated cardiomyopathy.

### Study limitations

A limitation of the present study is the use of a single iPSC line per group. Although the inclusion of multiple independent patient-derived lines would further strengthen the generalizability of the findings, this was partially mitigated using an isogenic CRISPR-corrected control line, which minimizes variability related to genetic background and allows for a more controlled assessment of the mutation-specific effects. Importantly, all experiments were performed across multiple independent differentiation batches and included repeated biological and technical replicates. The observed phenotypes were consistently reproduced across independent experiments, with low inter-batch variability. Collectively, these considerations support the robustness and reproducibility of the findings, despite the inherent limitation of using a single line per group. Finally, some of our findings are in line with previous results obtained in iPSC-CMs generated from *PRKAG2*-mutatn patients.

## Conclusion

In summary, this study offers valuable insights into the metabolic, mitochondrial and electrophysiological disruptions caused by *PRKAG2* mutations. Our findings reveal significant alterations in glycogen metabolism, glycolysis and lipid storage, combined with alterations in mitochondrial properties characterized by increased maximal respiratory capacity without changes in basal oxidative phosphorylation, all of which associated with the pathophysiology of *PRKAG2* cardiomyopathy. Notably, we identified metformin as a potential therapeutic approach, demonstrating its ability to modulate mitochondrial characteristics and reduce arrhythmias in mutant CMs. The selective action of metformin on mutant CMs, without affecting healthy or isogenic control CMs, emphasizes its promise as a targeted treatment. These beneficial effects are plausibly linked to modulation of AMPK-dependent pathways, although the precise molecular mechanisms remain to be determined. Further research, particularly in animal models, is necessary to confirm metformin therapeutic benefits and explore other interventions to address the complex metabolic and electrophysiological mechanisms underlying *PRKAG2* cardiomyopathy.

## Data Availability

The RNA-seq datasets generated and analyzed in this study are included in the Supplementary material, including normalized counts and differential expression analysis results. Additional data are available from the corresponding author upon reasonable request. The raw metabolomic and lipidomic data files are deposited in the MassIVE repository, accession number MSV000101195.

## References

[1] Arad M Maron BJ Gorham JM Johnson WH Saul JP Perez-Atayde AR Glycogen storage diseases presenting as hypertrophic cardiomyopathy. N Engl J Med. (2005) 352:362–72. 10.1056/NEJMoa033349 15673802

[2] Carling D. AMPK signalling in health and disease. Curr Opin Cell Biol. (2017) 45:31–7. 10.1016/j.ceb.2017.01.005 28232179

[3] Gwinn DM Shackelford DB Egan DF Mihaylova MM Vasquez DS Turk BE AMPK phosphorylation of raptor mediates a metabolic checkpoint. Mol Cell. (2008) 30:214–26. 10.1016/j.molcel.2008.03.003 18439900 PMC2674027

[4] Hardie DG Schaffer BE Brunet A. AMPK: an energy-sensing pathway with multiple inputs and outputs. Trends Cell Biol. (2016) 26:190–201. 10.1016/j.tcb.2015.10.013 26616193 PMC5881568

[5] Adams J Chen Z Van Denderen BJW Morton CJ Parker MW Witters LEEA Intrasteric control of AMPK via the *γ*_1_ subunit AMP allosteric regulatory site. Protein Sci. (2004) 13:155–65. 10.1110/ps.03340004 14691231 PMC2286513

[6] Gollob MH Green MS Tang ASL Gollob T Karibe A Hassan A-S Identification of a gene responsible for familial Wolff-Parkinson-White syndrome. N Engl J Med. (2001) 344:1823–31. 10.1056/NEJM200106143442403 11407343

[7] Ben Jehuda R Eisen B Shemer Y Mekies LN Szantai A Reiter I CRISPR correction of the PRKAG2 gene mutation in the patient’s iPSC-derived cardiomyocytes eliminates the electrophysiological and structural abnormalities. Heart Rhythm. (2017) 5:267–76. 10.1016/j.hrthm.2017.09.024 28917552

[8] Oliveira SMJ Ehtisham J Redwood CS Ostman-Smith I Blair EM Watkins H. Mutation analysis of AMP-activated protein kinase subunits in inherited cardiomyopathies: implications for kinase function and disease pathogenesis. J Mol Cell Cardiol. (2003) 35:1251–5. 10.1016/S0022-2828(03)00237-2 14519435

[9] Patel VV Arad M Moskowitz IPG Maguire CT Branco D Seidman JG Electrophysiologic characterization and postnatal development of ventricular pre-excitation in a mouse model of cardiac hypertrophy and Wolff-Parkinson-White syndrome. J Am Coll Cardiol. (2003) 42:942–51. 10.1016/S0735-1097(03)00850-7 12957447

[10] Blair E Redwood C Ashrafian H Oliveira M Broxholme J Kerr B Mutations in the *γ*2 subunit of AMP-activated protein kinase cause familial hypertrophic cardiomyopathy: evidence for the central role of energy compromise in disease pathogenesis. Hum Mol Genet. (2001) 10:1215–20. 10.1093/hmg/10.11.1215 11371514

[11] Cox GF. Diagnostic approaches to pediatric cardiomyopathy of metabolic genetic etiologies and their relation to therapy. Prog Pediatr Cardiol. (2007) 24:15–25. 10.1016/j.ppedcard.2007.08.013 19030119 PMC2585778

[12] Zou L Shen M Arad M He H Lofgren B Ingwall JS N488i mutation of the gamma-2-subunit results in bidirectional changes in AMP-activated protein kinase activity. Circ Res. (2005) 97:323–8. 10.1161/01.RES.0000179035.20319.c2 16051890

[13] Bridges HR Jones AJY Pollak MN Hirst J. Effects of metformin and other biguanides on oxidative phosphorylation in mitochondria. Biochem J. (2014) 462:475–87. 10.1042/BJ20140620 25017630 PMC4148174

[14] Feng J Wang X Ye X Ares I Lopez-Torres B Martínez M Mitochondria as an important target of metformin: the mechanism of action, toxic and side effects, and new therapeutic applications. Pharm Res. (2022) 177:106114. 10.1016/j.phrs.2022.106114 35124206

[15] Owen MR Doran E Halestrap AP. Evidence that metformin exerts its anti-diabetic effects through inhibition of complex 1 of the mitochondrial respiratory chain. Biochem J. (2000) 348:607–14. 10.1042/bj3480607 10839993 PMC1221104

[16] Wang Y An H Liu T Qin C Sesaki H Guo S Metformin improves mitochondrial respiratory activity through activation of AMPK. Cell Rep. (2019) 29:1511–1523.e5. 10.1016/j.celrep.2019.09.070 31693892 PMC6866677

[17] Novak A Barad L Lorber A Gherghiceanu M Rappaport B. Functional abnormalities in iPSC-derived cardiomyocytes generated from CPVT1 and CPVT2 patients carrying ryanodine or calsequestrin mutations. J Cell Mol Med. (2015) 19:2006–18. 10.1111/jcmm.12581 26153920 PMC4549051

[18] Yehezkel S Rebibo-Sabbah A Segev Y Tzukerman M Shaked R Huber I Reprogramming of telomeric regions during the generation of human induced pluripotent stem cells and subsequent differentiation into fibroblast-like derivatives. Epigenetics. (2011) 6:63–75. 10.4161/epi.6.1.13390 20861676 PMC3052915

[19] Banerjee SK Ramani R Saba S Rager J Tian R Mathier MA A PRKAG2 mutation causes biphasic changes in myocardial AMPK activity and does not protect against ischemia. Biochem Biophys Res Commun. (2007) 360:381–7. 10.1016/j.bbrc.2007.06.067 17597581

[20] MacKay GM Zheng L Van Den Broek NJF Gottlieb E. Analysis of cell metabolism using LC-MS and isotope tracers. Methods Enzymol. (2015) 561:171–96. 10.1016/bs.mie.2015.05.016 26358905

[21] Ben-Ari M Schick R Barad L Novak A Ben-Ari E Lorber A From beat rate variability in induced pluripotent stem cell-derived pacemaker cells to heart rate variability in human subjects. Heart Rhythm. (2014) 11:1808–18. 10.1016/j.hrthm.2014.05.037 25052725 PMC4283811

[22] Mandel Y Weissman A Schick R Barad L Novak A Meiry G Human embryonic and induced pluripotent stem cell-derived cardiomyocytes exhibit beat rate variability and power-law behavior. Circulation. (2012) 125:883–93. 10.1161/CIRCULATIONAHA.111.045146 22261196 PMC3697086

[23] Shemer Y Mekies LN Ben Jehuda R Baskin P Shulman R Eisen B Investigating LMNA-related dilated cardiomyopathy using human induced pluripotent stem cell-derived cardiomyocytes. Int J Mol Sci. (2021) 22:7874. 10.3390/ijms22157874 34360639 PMC8346174

[24] Linnett PE Beechey RB. Inhibitors of the ATP synthetase systems. Methods Enzymol. (1979) 55:472–518. 10.1016/0076-6879(79)55061-7 156854

[25] Heytler PG Prichard WW. A new class of uncoupling agents—carbonyl cyanide phenylhydrazones. Biochem Biophys Res Commun. (1962) 7:272–5. 10.1016/0006-291X(62)90189-4 13907155

[26] Tzagoloff A. Oxidative phosphorylation. In: Siekevitz P, editor. Mitochondria. New York, NY: Springer (1982). p. 131–56.

[27] Luptak I Shen M He H Hirshman MF Musi N Goodyear LJ Aberrant activation of AMP-activated protein kinase remodels metabolic network in favor of cardiac glycogen storage. J Clin Invest. (2007) 117:1432–9. 10.1172/JCI30658 17431505 PMC1847536

[28] Banankhah P Fishbein GA Dota A Ardehali R. Cardiac manifestations of PRKAG2 mutation. BMC Med Genet. (2018) 19:1. 10.1186/s12881-017-0512-6 29298659 PMC5751825

[29] Travis Hinson J Chopra A Lowe A Sheng CC Gupta RM Kuppusamy R Integrative analysis of PRKAG2 cardiomyopathy iPS and microtissue models identifies AMPK as a regulator of metabolism, survival, and fibrosis. Cell Rep. (2016) 17:3292–304. 10.1016/j.celrep.2016.11.066 28009297 PMC5193246

[30] Zhan Y Sun X Li B Cai H Xu C Liang Q Establishment of a PRKAG2 cardiac syndrome disease model and mechanism study using human induced pluripotent stem cells. J Mol Cell Cardiol. (2018) 117:49–61. 10.1016/j.yjmcc.2018.02.007 29452156

[31] Stefan Buré A Gomes AL Teijeiro A Campos-Olivas R Megías D Buré S Regulation of OGT by URI in response to glucose confers c-MYC-dependent survival mechanisms. Cancer Cell. (2016) 30:290–307. 10.1016/j.ccell.2016.06.023 27505673

[32] Siasos G Tsigkou V Kosmopoulos M Theodosiadis D Simantiris S Tagkou NM Mitochondria and cardiovascular diseases—from pathophysiology to treatment. Ann Transl Med. (2018) 6:256. 10.21037/atm.2018.06.21 30069458 PMC6046286

[33] Liu X Wang S Guo X Li Y Ogurlu R Lu F Increased reactive oxygen species-mediated Ca^2+^/calmodulin-dependent protein kinase II activation contributes to calcium handling abnormalities and impaired contraction in Barth syndrome. Circulation. (2021) 143:1894–911. 10.1161/CIRCULATIONAHA.120.048698 33793303 PMC8691127

[34] Zhang J Chou OHI Tse YL Ng KM Tse HF. Application of patient-specific iPSCs for modelling and treatment of X-linked cardiomyopathies. Int J Mol Sci. (2021) 22:8132. 10.3390/ijms22158132 34360897 PMC8347533

[35] Pabel S Knierim M Stehle T Alebrand F Paulus M Sieme M Effects of atrial fibrillation on the human ventricle. Circ Res. (2022) 130:994–1010. 10.1161/CIRCRESAHA.121.319718 35193397 PMC8963444

[36] Sacchetto C Sequeira V Bertero E Dudek J Maack C Calore M. Metabolic alterations in inherited cardiomyopathies. J Clin Med. (2019) 8:2195. 10.3390/jcm8122195 31842377 PMC6947282

[37] Leverve XM Guigas B Detaille D Batandier C Koceir EA Chauvin C Mitochondrial metabolism and type-2 diabetes: a specific target of metformin. Diabetes Metab. (2003) 29:6S88–94. 10.1016/S1262-3636(03)72792-X 14502105

[38] Bharath LP Agrawal M McCambridge G Nicholas DA Hasturk H Liu J Metformin enhances autophagy and normalizes mitochondrial function to alleviate aging-associated inflammation. Cell Metab. (2020) 32:44–55.e6. 10.1016/j.cmet.2020.04.015 32402267 PMC7217133

[39] Zhang LP Hui B Gao B-R. High risk of sudden death associated with a PRKAG2-related familial Wolff-Parkinson-White syndrome. J Electrocardiol. (2011) 44:483–6. 10.1016/j.jelectrocard.2010.02.009 20381067

[40] Lang F Föller M. Regulation of ion channels and transporters by AMP-activated kinase (AMPK). Channels. (2014) 8:20–8. 10.4161/chan.27423 24366036 PMC4048339

